# Gene, Protein, and in Silico Analyses of FoxO, an Evolutionary Conserved Transcription Factor in the Sea Urchin *Paracentrotus lividus*

**DOI:** 10.3390/genes15081078

**Published:** 2024-08-15

**Authors:** Roberta Russo, Maria Antonietta Ragusa, Walter Arancio, Francesca Zito

**Affiliations:** 1 Istituto per la Ricerca e l’Innovazione Biomedica (IRIB), Consiglio Nazionale delle Ricerche, Via Ugo La Malfa 153, 90146 Palermo, Italy; walter.arancio@irib.cnr.it (W.A.); francesca.zito@irib.cnr.it (F.Z.); 2 Dipartimento di Scienze e Tecnologie Biologiche, Chimiche e Farmaceutiche (STEBICEF), Università degli Studi di Palermo, Viale delle Scienze Ed. 16, 90128 Palermo, Italy; maria.ragusa@unipa.it

**Keywords:** development, mRNA expression, FH domain, FoxO protein interactions, sea urchin, potential therapeutics

## Abstract

FoxO is a member of the evolutionary conserved family of transcription factors containing a Forkhead box, involved in many signaling pathways of physiological and pathological processes. In mammals, mutations or dysfunctions of the *FoxO* gene have been implicated in diverse diseases. FoxO homologs have been found in some invertebrates, including echinoderms. We have isolated the FoxO cDNA from the sea urchin *Paracentrotus lividus* (*Pl-foxo*) and characterized the corresponding gene and mRNA. In silico studies showed that secondary and tertiary structures of *Pl-foxo* protein corresponded to the vertebrate FoxO3 isoform, with highly conserved regions, especially in the DNA-binding domain. A phylogenetic analysis compared the *Pl-foxo* deduced protein with proteins from different animal species and confirmed its evolutionary conservation between vertebrates and invertebrates. The increased expression of *Pl-foxo* mRNA following the inhibition of the PI3K signaling pathway paralleled the upregulation of *Pl-foxo* target genes involved in apoptosis or cell-cycle arrest events (*BI-1*, *Bax*, *MnSod*). In silico studies comparing molecular data from sea urchins and other organisms predicted a network of *Pl-foxo* protein–protein interactions, as well as identified potential miRNAs involved in *Pl-foxo* gene regulation. Our data may provide new perspectives on the knowledge of the signaling pathways underlying sea urchin development.

## 1. Introduction

Transcription factors (TFs) are very important key molecules in the complex networks that regulate cellular homeostasis and signaling pathways during embryogenesis or adult life in all living organisms. TFs are grouped in large families according to the structures of their DNA-binding domains, such as basic Leucine Zipper (bZIP), Zinc Finger (Znf), homeodomain Leucine Zipper (HD-Zip), Rel-homology-domain (RHD), Forkhead box (Fox), Nuclear Hormone Receptor (NHR) and Helix–Loop–Helix (HLH) [1].

Fox proteins are members of the evolutionarily ancient Forkhead box family that was first identified in *Drosophila* with a mutation that produced head structures like a fork. Fox proteins are also referred to as ‘winged helix’ proteins because the 3D structure of their DNA-binding domain, the Forkhead box (110 amino acids long), obtained by X-ray crystallography, showed a core of three α-helices and three antiparallel β-strands, flanked by two loops, similar, indeed, to the butterfly wings [2]. Since the first discovery, hundreds of Fox genes have been found in different species, which were recently classified in 19 subfamilies from FoxA to FoxS on the basis of similarities and evolution in their unifying feature, i.e., the highly conserved DNA-binding domain. Originating in unicellular eukaryotes, Fox genes have spread, either through multiple duplication or gene loss events, throughout the animal kingdom, yet evolved distinct roles [3]. FoxO factors belong to the Forkhead “O” subfamily, closely related to FoxM and FoxP, and, differently from other Fox subfamilies, they are evolutionarily conserved mediators of insulin and growth factor signaling. These proteins can play many roles, such as the regulation of development, apoptosis, cell death, oxidative stress resistance, longevity, glucose metabolism, cell cycle, cell differentiation, and cancer [4].

FoxO TFs are regulated overall by multiple mechanisms: at the post-transcriptional level, by non-coding RNAs (e.g., miRNAs) or other co-factors, and at the post-translational level by phosphorylation, acetylation, methylation and ubiquitination. Phosphorylation is the major regulator of FoxO TFs activities, controlling their nuclear-cytoplasmic shuttling. In particular, the most important positive regulator is c-Jun N-terminal kinase (JNK), which phosphorylates FoxOs under stress conditions [5]. On the contrary, FoxO TFs are negatively regulated by the PI3K/Akt signaling pathway in response to external and internal stimuli [4]. Post-transcriptional regulation of FoxO expression occurs mainly by numerous miRNAs in response to stressful stimuli, including oxidative stress and cancer [6].

Among invertebrates, FoxO orthologs have been identified in Hydra [7], Caenorhabditis elegans [8], Drosophila [9], and in the sea urchin Strongylocentrotus purpuratus [10]. In invertebrates, there is usually only one FoxO gene, which is different from humans, where there are four: FoxO1 (previously FKHR), FoxO3 (FKHRL1, alias FoxO2), FoxO4 (MLLT7, alias AFX), and FoxO6 [11].

The availability of the annotated genome of the sea urchin *S. purpuratus* [12] has allowed the systematic identification of all TF families [13,14,15], including a Fox with almost all but four subfamily members. In *S. purpuratus*, FoxA, FoxB and FoxN2/3 have been well described [16,17,18], while the FoxO gene (Sp-foxo1 in Echinobase database, https://www.echinobase.org/echinobase/, accessed on 15 January 2024) was only described for its embryonic expression by Tu et al. [10], but its function has not yet been addressed.

Recently, the *Paracentrotus lividus* genome has also been completed, even if it is not yet fully available and freely usable [19].

The *P. lividus* species has been used for many years as a sentinel of marine environmental stress, and is a valid tool for discovering the molecular and regulatory mechanisms underlying both the adult immune system and embryogenesis [20,21,22]. In addition, the sea urchin occupies an important phylogenetic position, being a deuterostome like vertebrates, and thus representing a strong evolutionary link between invertebrates and vertebrates.

Given the complexity of the FoxO proteins, being able to study them in a simple model system but one that is evolutionarily related to vertebrates, such as the sea urchin, could facilitate the acquisition of new knowledge about both their regulation and their role in more evolved organisms.

Here, we report the study of a member of the TF FoxO family, referred to as *Pl-foxo*, from the sea urchin *P. lividus*, and the characterization of the gene, mRNA and predicted protein. We describe the phylogenetic relationship among *Pl-foxo* and homologues proteins from sea urchin *S. purpuratus* as well as other invertebrate and vertebrate organisms. We determined the temporal expression profile of the *Pl-foxo* mRNA during *P. lividus* embryonic development and analyzed its expression in PI3K-inhibited embryos, together with some of its regulatory and target genes. In addition, we carried out in silico analyses to predict interaction networks of the sea urchin FoxO protein and to identify potential regulatory miRNAs.

## 2. Materials and Methods

### 2.1. Sampling of Animals

Gametes were collected from adult sea urchins of *P. lividus*, fished in the northwestern coast of Sicily in the Mediterranean Sea. Eggs were fertilized and embryos were grown in Millipore (Billerica, MA, USA, 0.22 μm) filtered sea water (MFSW), containing antibiotics (50 mg/1 streptomycin sulfate and 30 mg/1 penicillin), at the dilution of 4000 embryos/mL and at the moderate temperature of 18 ± 1 °C.

### 2.2. Preparation of PI3K-Inhibited Embryos

Embryos were prepared according to the methods of Chiaramonte et al. [23]. Briefly, inhibition of PI3K activity was performed using LY294002 (LY, Sigma Chemical Co., St. Louis, MO, USA), dissolved in dimethylsulfoxide (DMSO, Sigma Chemical Co., St. Louis, MO, USA), at the final concentration of 40 μM. Embryos were continuously cultured in the presence of LY from the blastula stage (16 h post-fertilization), in 24-multiwell plates (Cellstar, Greiner Bio-One GmbH, Kremsmünster, Austria), (2.5 × 10^3^ embryos/mL/well), at 18 °C in the dark. Control embryos were cultured in the presence of equivalent concentrations of DMSO as those used for LY-treated embryos. Control and LY-treated embryos were collected 48 h (64 h post-fertilization) after LY addition and stored at −20 °C as pellets until use.

### 2.3. RNA Extraction, cDNA Synthesis, Cloning, Sequencing and cDNA Analysis

Total RNAs from gastrula, control and LY-treated embryos was extracted using “GenElute Mammalian Total RNA Miniprep Kit” according to the manufacturer’s instructions (Sigma Chemical Co., St Louis, MO, USA) and quantified using a bio-photometer (Eppendorf S.r.l., Hamburg, Germany). Before synthesizing cDNA, we tested the obtained RNAs by PCR with primers for the *Pl-Z12-1* reference gene to ensure no genomic contamination. Total RNAs (1 μg) were reverse transcribed according to the manufacturer’s instructions (Applied Biosystems, Life technologies, Carlsbad, CA, USA). An aliquot of the achieved cDNA (20 ng) was used to perform Polymerase Chain Reaction (PCR), using different primers designed on the *Sp-FoxO1* sequence to obtain overlapping fragments of cDNA. The amplicons obtained from the PCR were cloned in the pGEM-Teasy vector, following the Promega manufacturer instruction manual (Promega, Madison, WI, USA) and sequenced by a service company (BIO-FAB research srl, Rome, Italy). The overlapping sequence fragments were assembled in a unique sequence, named *Pl-foxo*, which has been deposited at NCBI (http://www.ncbi.nlm.nih.gov/, accessed on 22 March 2024) under the accession number MT799801.2.

Using this complete *Pl-foxo* sequence as a query in the *P. lividus* expressed sequence tags (EST) database, similar sequences were retrieved via BLAST (https://blast.ncbi.nlm.nih.gov/Blast.cgi, accessed on 12 August 2024). Additionally, transcriptomic reads from the Sequence Read Archive (SRA—https://www.ncbi.nlm.nih.gov/sra, accessed on 18 January 2024) and from a specific *P. lividus* database (Supplementary File S1) were also retrieved. These sequences were assembled using CAP3 (https://doua.prabi.fr/software/cap3, accessed on 18 January 2024), and the resulting contig consensus sequence was verified through a BLAST search. The cDNA sequence was translated using ORF Finder (https://www.ncbi.nlm.nih.gov/orffinder/, accessed on 22 January 2024).

### 2.4. Gene Annotation

The genomic region of interest was identified by performing a BLAST search using the contig corresponding to the *Pl-foxo* sequence as a query in the whole-genome shotgun contigs database of *P. lividus* (GenBank assemblies GCA_940671915.1, submitted by the University of Oxford, Oxford, UK, and GCA_033220595.1, submitted by Stazione Zoologica Anton Dohrn, Naples, Italy) [19,24]. The initial gene model was created using the ab initio gene annotation tool FGENESH, selecting sea urchin-specific gene-finding parameters (http://www.softberry.com/berry.phtml?topic=fgenesh&group=programs&subgroup=gfind, accessed on 6 February 2024). The gene model was then manually edited by integrating transcript alignment data. The accuracy of the annotation was corroborated by comparing the sequence resulting from the FGENESH annotation with available homologs. The predicted mRNA sequence was verified by comparing it with the transcript sequence using Needle [25]. The same tool was also used to compare the predicted protein sequences. Clustal Omega (https://www.ebi.ac.uk/jdispatcher/msa/clustalo, accessed on 6 February 2024) was used to align the three ORFs from the gene annotation, the clone and the contig nucleotide sequences and deduced amino acid sequences.

A footprinting approach has been employed to detect the most likely occurrences of TF binding events in the regulatory region of *Pl-foxo* (RNA-seq and ATAC-seq signal) as described in Marlétaz et al. [19].

### 2.5. Quantitative Expression of Pl-foxo (qPCR)

Quantification of gene expression was performed by using the StepOnePlus real-time qPCR, as described in the manufacturer’s manual (Applied Biosystems) with a Comparative Threshold Cycle Method using SYBR Green chemistry [26]. The *Pl*-*Z12-1* mRNA was used as a reference gene [27]. The qPCR was run as follows: 1× cycle denaturing at 95 °C for 10′ for DNA polymerase activation; 38× cycles: melting at 95 °C for 15″, annealing/extension at 60 °C for 60″. *Pl-foxo* primers were reported in Russo et al. [28]. *Pl-pi3k* primers were reported in Chiaramonte et al. [23]. *Pl-Bi-1* primers included Forward: 5′TCTCAGTGGAAACCGGAAAGT3′; Reverse: 5′TTGACATAGCTTCCAACTGCA3′ (Accession: PP731550); *Pl-bax* primers included Forward: 5′TCCTTGTGATGAAACTGATGCAT3′; Reverse: 5′ACAATGAAATGTTGAAGGAGAA3′ (Accession: HG931725.1); *Pl-mnsod* primers included Forward: 5′TAAGGAGCCAAGCCAGAGTGGTT3′; Reverse: 5′AAGCTCCATGATCTCACTGCTAA3′ (Accession: PP731551).

### 2.6. Statistical Analysis

Statistical analysis was performed on qPCR values obtained from at least three independent experiments using the Excel *T*-test, with considered significance at *p* < 0.05.

### 2.7. In Silico Analyses of the Protein, Phylogenetics, and miRNA Prediction

Homologs of the proteins from different organisms, belonging to both invertebrate and vertebrate phyla, have been obtained by the BLAST program (http://blast.ncbi.nlm.nih.gov/, accessed on 23 March 2024) and their Accession numbers are indicated in Table 1.

The Conserved Domain Database (CDD) was used to predict the conserved domains that characterize the *Pl-foxo* TF [29]. Protein multiple alignments of the obtained proteins were performed by ClustalW2.1 (https://www.genome.jp/tools-bin/clustalw, accessed on 25 March 2024) and by the Boxshade program at the https://junli.netlify.app/apps/boxshade/ (accessed on 25 March 2024) site. Phylogenetic reconstructions were performed using the function “build” of ETE3 3.1.2 (GenomeNet https://www.genome.jp/tools/ete/, accessed on 26 March 2024). Alignment was carried out with Clustal Omega v1.2.4 with the default options. The ML tree was inferred using PhyML v20160115 [30]. Predictions of secondary structure models of the *Pl-foxo* protein were obtained by the Phyre2 web portal for protein modeling, prediction and analysis [31]. The tertiary structure of the *Pl-foxo* protein was found by using the software I-Tasser (https://zhanglab.ccmb.med.umich.edu/I-TASSER, accessed on 9 April 2024). The software Netphos 3.1 (https://services.healthtech.dtu.dk/service.php?NetPhos-3.1, accessed on 10 April 2024), an artificial network method that predicts phosphorylation sites, was used to predict the phosphorylation sites (with high scores above 0.8 for T and Y and 0.9 for S). Ubiquitination prediction was found using the tool RUBI 1.0 at the site: http://old.protein.bio.unipd.it/rubi/, accessed on 12 August 2024. Information about the protein was found in the Protein Data Bank, PDB, at https://www.rcsb.org/, accessed on 12 August 2024.

The predicted protein–protein interactions were obtained via the STRING database (http://string-db.org/, accessed on 18 June 2024) in four different organisms, i.e., *S. purpuratus* (*P. lividus* proteins are not annotated in the STRING database), *C. elegans*, *D. melanogaster*, and *H. sapiens*. In detail, we simulated two different protein–protein networks for each organism, i.e., searching for the predicted interactions between FoxO and all the proteins encoded by the genes analyzed by real-time qPCR in the PI3K-inhibited embryos, i.e., Bax, BI-1, PI3K, and Mnsod, together with Akt, Sirt1 and 14.3.3e (confidence interaction score 0.300), or searching for all predicted interactions for FoxO proteins (Sp-foxo1 and homologs) in the four organisms, setting the highest confidence interaction score (0.900). The network analyses were performed taking into consideration all the seven “evidence channels”, i.e., the active interaction sources, including known (databases, experiments) and predicted (gene neighborhood, fusion, co-occurrence) interactions, as well as text mining (proteins that are frequently mentioned together), co-expression and protein homology.

To identify miRNA–target interactions for human FoxO3 transcripts, we used miRTarBase (https://mirtarbase.cuhk.edu.cn/, accessed on 12 August 2024), a database that collects experimentally validated microRNA–target interactions and PITA (https://tools4mirs.org/software/target_prediction/pita/, accessed on 15 April 2024). High-confidence predicted miRNA binding sites for the 3′UTR of the main human FoxO3 transcript (ENST00000343882) were retrieved using the MBS database [32]. The MBS database reports miRNA binding sites predicted by three widely used prediction algorithms, with the following parameters: miRanda (https://tools4mirs.org/software/target_prediction/miranda/, accessed on 16 April 2024): a Binding energy ≤ −20 kcal/mol and a score ≥ 140; PITA: ΔΔE ≤ −10 kcal/mol; TargetScan 8.0 (https://www.targetscan.org/vert_80/, accessed on 16 April 2024): Binding site type = 8mer-1a, 7mer-1a, or 7mer-m8. The 3′UTR of *Pl-foxo* mRNA was analyzed looking for putative human miRNA binding sites using miRanda at a Binding energy ≤ −20 kcal/mol and a score ≥ 140 and PITA at ΔΔE ≤ −10 kcal/mol. mirBASE was accessed at the site https://mirbase.org/, accessed on 12 August 2024.

## 3. Results

### 3.1. Characterization of Pl-foxo cDNA

In this study, we isolated and sequenced the complete cDNA open reading frame (ORF), named *Pl-foxo*, from *P. lividus* late gastrula embryos. The *Pl-foxo* ORF is 1656 not long and codes for a protein of 551 amino acids. Moreover, using this isolated *Pl-foxo* sequence as a query in the *P. lividus* databases, several sequences containing the *Pl-foxo* ORF were retrieved. The cDNA sequences were assembled into a contig that resulted in being 2757 nucleotides long (Figure S1a). The predicted ORF starts at nucleotide 182 and stops at nucleotide 1837. The isolated cDNA showed a high identity (86.8%) with the *S. purpuratus* (*Sp-foxo1*) transcript ORF.

### 3.2. Gene Analysis of Pl-foxo

**Figure 1:**
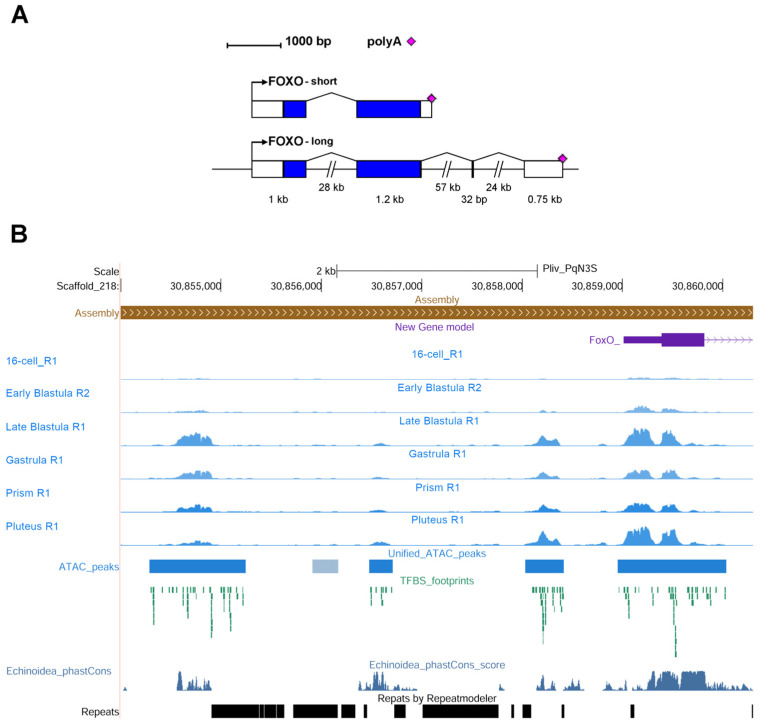
*Pl-foxo* gene analysis. (**A**) Intron–exon structure of the *Pl-foxo* gene showing the two possible alternative splicings that result in short and long mRNA isoforms. Rectangles represent exons: white rectangles represent 5′UTR and 3′UTR sequences, and colored rectangles represent coding sequences. Numbers below the black lines and rectangles indicate the size (kb/bp) of the corresponding introns or exons, respectively. Pink diamonds represent the putative polyadenylation sites. (**B**) The regulatory region around the *Pl-foxo* gene promoter is visualized as tracks in the UCSC genome browser. The first exon of *Pl-foxo* is shown in purple, ATAC-seq signals and peaks are shown in blue, transcription factor binding sites (TFBS) are in green, conserved sequences in Echinoidea are in dark blue and repeats are in black.

We also studied the gene structure of *Pl-foxo* and its regulatory region. The *Pl-foxo* gene is located on Scaffold 218 of the current *P. lividus* genome assembly (Pliv_v1) and spans a genomic distance of 112 kb. It comprises four exons and three introns, but only the largest exons, 1 and 2, which are 1 kb and 1.2 kb long, respectively, encode the protein (Figure 1A). The third exon is only 32 bp long, and the fourth is approximately 750 bp long. Two polyadenylation sites were predicted, which are compatible with two mRNA isoforms that code for the same protein. An unspliced exon 2–intron 2 transcript could generate a short transcript isoform with a different 3′UTR than the long isoform. A new search in transcript databases confirmed this hypothesis and the sequence of the short isoform of the transcript is shown in Figure S1b.

Exon–intron boundary sequences reported in Table 2 show that all introns conform to the GT-AG splicing rule. The alignment of the coding sequences from the cDNA and the gene annotation showed 99.0% identity.

Furthermore, the predicted protein sequence comparison highlights two differences (polymorphisms), the first of which is conservative (375 S→N; 378 N→G) (yellow in Figure S2). Figure S2 shows the multiple sequence alignment of *Pl-foxo* ORF nucleotide sequences from the gene annotation, the cDNA clone and the cDNA contig.

The footprint approach described in Marlétaz et al. [19] was used to identify the TF binding events in the regulatory region of *Pl-foxo* (Figure 1B). The putative regulatory region considered (Scaffold_218:30,854,000–30,860,300) contains four ATAC-seq (Assay for Transposase-Accessible Chromatin using sequencing) peaks with binding sites for 88 transcription factors. The fourth region is near to the putative Transcription Start Site (TSS) and contains the proximal promoter (Table 3).

### 3.3. In Silico Protein Analysis

The deduced *Pl-foxo* protein, which is 551 aa long, has a theoretical isoelectric point (pI) of 6.55 and an estimated molecular mass of 61 kDa. We compared the isolated protein sequence with the FoxO protein sequences from several organisms included in the NCBI database. In Table 1, we report the similarity percentages, with respect to the number of overlapping amino acids. In particular, *Pl-foxo* showed a high percentage of similarity (94%) with the FoxO1 from the sea urchins *S. purpuratus* (Sp-foxo1) and *L. variegatus* (FoxO1-like), and a lower percentage of similarity (ranging from 57 to 67%) with other echinoderms. The *Pl-foxo* protein showed about 50% similarity with its orthologs in chordates/vertebrates. In addition, the percentages of similarity were 62% with FoxO from the insect *D. melanogaster* and 63% with *C. elegans*.

**Figure 2:**
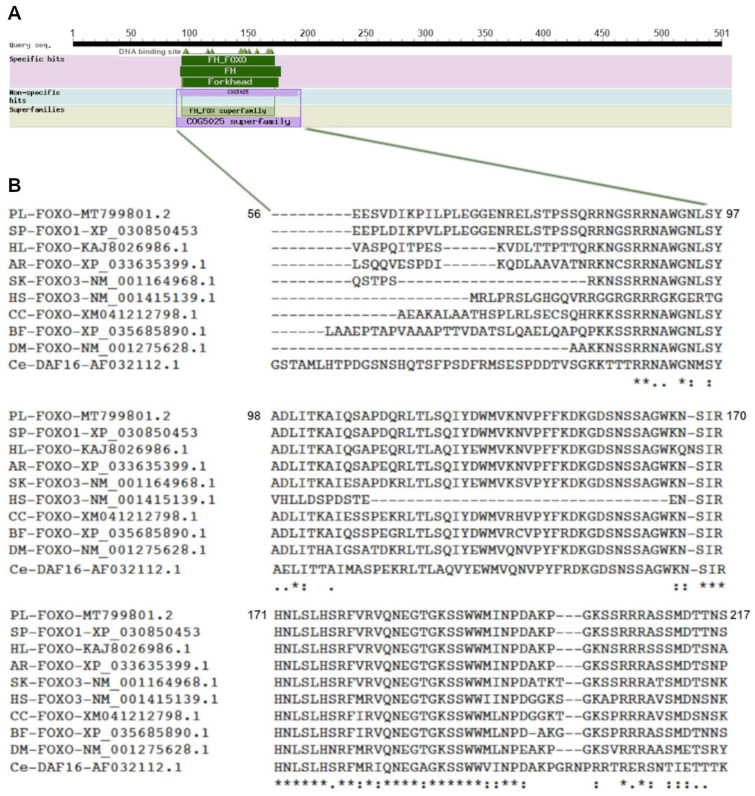
*Pl-foxo* protein analysis. (**A**) Diagram of the *Pl-foxo* protein obtained by Blast program, showing the most conserved regions of the FoxO superfamily and specific regions. (**B**) Clustal W alignment of the Forkhead DNA-binding domain of FoxO proteins from different organisms: Pl, *P. lividus*; Sp, *S. purpuratus*; Hl, *H. leucospilota*; Ar, *A. rubens*; Sk, *S. kowaleskii*; Hs, *H. sapiens*; Cc, *C. charcarias*; Bb, *B. floridae*; Dm, *D. melanogaster*; Ce, *C. elegans*. Asterisk = fully conserved amino acids; colon = conservative substitutions; period = semi-conservative substitutions.

The protein sequence of *Pl-foxo* was analyzed in order to predict the conserved domains that characterize the FoxO family of DNA-binding TFs (Figure 2A). The protein was identified as the FH_FoxO: Forkhead (FH) domain FoxO subfamily. Figure 2B shows the sequence alignment of DNA-binding domains (DBDs) from FoxO proteins derived from diverse species of invertebrates (insects, echinoderms), cephalochordates and vertebrates, obtained by Clustal W alignment (Figure S3).

Figure S4 reports a boxshade analysis of the FoxO proteins’ alignment that puts in evidence the perfect identity of the amino acids or the conservative substitutions with black and gray boxes, respectively. Very high protein sequence similarity concerned the region where the DBD lies (from 90 to 225 aa), while other smaller regions of high similarity are scattered throughout the FoxO proteins (from 1 to 30 aa; from 230 to 275 aa).

**Figure 3:**
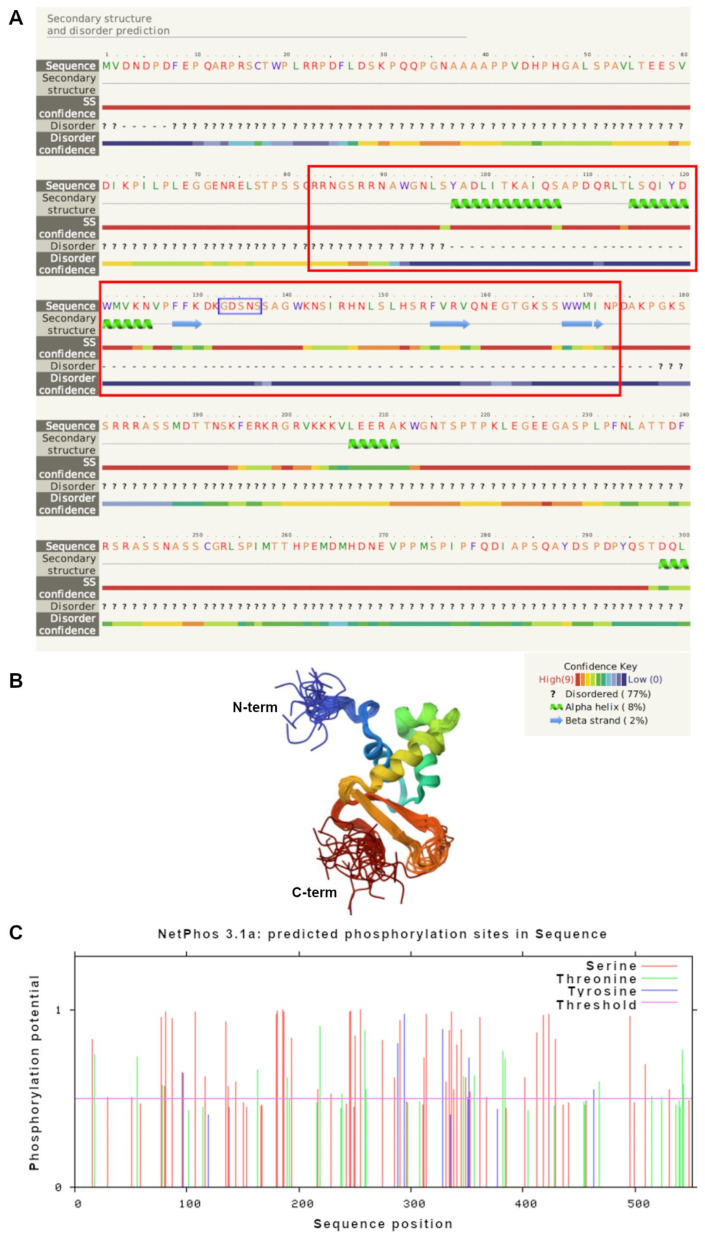
A 2/3D analysis of *Pl-foxo*. (**A**) The secondary structure of the DNA-binding domain of *Pl-foxo* transcription factor obtained by the Phyre2 program: the Forkhead domain is highlighted with two red rectangles. (**B**) The 3D structure predicted for *Pl-foxo* DBD obtained by I-Tasser software. (**C**) Scheme of the phosphorylation sites and their positions inside the *Pl-foxo* protein, revealed by the Netphos program 3.1.

Figure 3A shows the predicted secondary structure of the N-terminal of *Pl-foxo* (within M1 to L300 amino acids), which includes the DBD, obtained by Phyre 2 software. A total of 77% of the protein has coiled coil (disordered) domains, 8% has α helices (H), and 2% has β sheets (S). The complete predicted secondary structure is shown in Figure S5.

*Pl-foxo* DBD is restricted to 88 amino acids (from R86 to P173, red rectangles in Figure 3A), including two main H (Y97-S107 and L115-N125 amino acids) and three S (F128-D131, F155-Q159 and W168-N172 amino acids). The core amino acids sequence GDSNS of the DBD (blue rectangle in Figure 3A) is located at 133-137aa, before the last two serine.

The image of Figure 3B is a 3D model predicted for the *Pl-foxo* DBD, constructed on the template 2K86, which is based on the crystal structure of Foxo3a DBD from *H. sapiens* (doi: https://doi.org/10.2210/pdb2K86/pdb, accessed on 12 August 2024), with the best score (83%). In blue, the N-terminal is shown, and in red, the C-terminal of the protein. The two wings (W) are disordered sequences.

A further analysis of the entire *Pl-foxo* protein sequence has been performed by means of the Netphos program. Many predicted phosphorylation sites were found in the *Pl-foxo* protein: 19 serines (S); 2 threonines (T); 3 tyrosines (Y) (Figure 3C). In addition, we evaluated if these predicted phosphorylation sites were conserved among the invertebrate and vertebrate organisms previously considered. This analysis highlighted that only some of the 24 predicted most probable phosphorylation sites were greatly conserved in both vertebrates and invertebrates, such as S87, S135, S187, S246, and S255. Other phosphorylation residues were conserved only among sea urchins, such as T219, T259, S77, S81, S290, S314, S418, S423, S495, and Y294, and all others remaining ones were conserved only in invertebrates. Y328 is conserved in all echinoderms. Figure S3 shows phosphorylation sites highlighted in red. The ubiquitination prediction revealed no ubiquitinated lysines out of the 19 total lysines.

By the ProtParam site at Expasy, we deduced other information about the *Pl-foxo* protein. For example, it is enriched with the amino acids of prolin (10.5%) and serine (11.4%) and is poor in cysteine (0.7%). The total number of negatively charged residues (asparagine and glutamine) is 57, while the total number of positively charged residues (arginine and lysine) is 51. The estimated half-life of the *Pl-foxo* protein is 30 h. The instability index is computed to be 69.89, which classifies the protein as unstable.

### 3.4. Phylogenetic Analysis of FoxO Proteins

**Figure 4:**
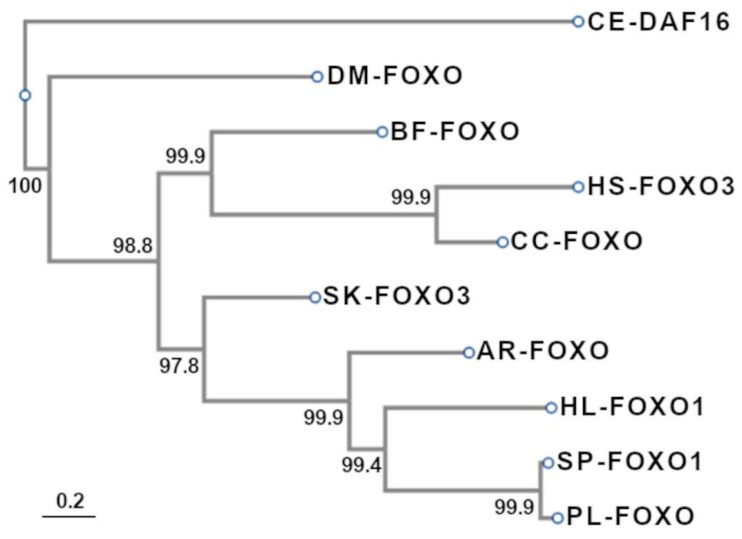
Evolutionary relationships of taxa for the *Pl-foxo* protein. Evolutionary analyses were conducted in ETE3 3.1.2. The tree is drawn to scale, with branch lengths proportional to the evolutionary distances used to infer the phylogenetic tree. The analysis involved 10 amino acid sequences. Ar, *A. rubens*, Bb, *B. floridae*; Dm, *D. melanogaster*; Cc, *C. charcarias*; Ce, *C. elegans*; Hl, *H. leucospilota*; Hs, *H. sapiens*; Pl, *P. lividus*; Sp, *S. purpuratus*; Sk, *S. kowaleskii*.

To construct the phylogenetic tree, we used the Clustal W program to align *Pl-foxo* with the protein sequences from the organisms indicated in Table 1 (see alignment in Figure S3). The Daf-16 protein of *C. elegans* was used as an outgroup sharing a common ancestor with the other organisms analyzed here. The phylogenetic tree is shown in Figure 4. As expected, *Pl-foxo* clusters in the same clade with the sea urchin Sp-foxo1, confirming their major similarity found by the alignment program, and together they cluster with the FoxO proteins from the other echinoderms analyzed, i.e., *A. rubens* and *H. leucospilota.* The FoxO protein from *D. melanogaster* forms a clade on its own. The FoxO3 protein from the hemichordate *S. kowaleskii* forms a cluster with the *B. floridae* protein as well as with the two FoxO proteins of the vertebrates analyzed, namely the shark *Carcharodon carcharias* and *H. sapiens*. Figure S6 is extremely interesting as it shows another phylogenetic tree of FoxO proteins, that gives us a broader vision. In fact, many different organisms have been analyzed here that show the best score with *P. lividus*, most of them being marine invertebrates, such as mussels, oysters, clams and hemichordates, all of them clustering independently of each other. The vertebrate fish clades are clustered at the top of the tree, while the *Pl-foxo* protein forms a specific clade with the FoxO proteins from the sea cucumber and other echinoderms at the bottom of the tree (highlighted in green).

### 3.5. Temporal Expression of Pl-foxo in the P. lividus Embryos during Development

**Figure 5:**
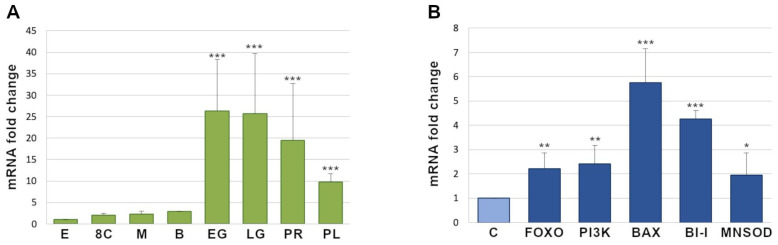
qPCR analyses of the *Pl-foxo* transcription levels in normal or PI3K-inhibited *P. lividus* embryos. (**A**) *Pl-foxo* expression levels throughout *P. lividus* sea urchin embryogenesis: E, Eggs (used as reference samples and assumed as 1 in the histogram); 8C, 8 cells; M, morula; B, blastula; EG, early gastrula; LG, cate gastrula; Pr, prism; Pl, pluteus. Mean values were significantly different. The asterisks (*) indicate statistically significative variations to the relative reference sample. Each bar represents the mean of three independent experiments ± SD (* *p* < 0.05, ** *p* < 0.01, *** *p* < 0.001). (**B**) Expression of *PI3K* and *FoxO* mRNAs, in addition to target genes of *Pl-foxo* (*Pl-Bi-1*, *Pl-Bax*, *Pl-MnSod*) in PI3K-inhibited embryos.

By qPCR, we studied the temporal expression of *Pl-foxo* mRNA at different developmental stages during sea urchin embryogenesis (Figure 5A). *Pl-foxo* mRNA was upregulated at all the developmental stages analyzed. In particular, an increase of 2.05-, 2.27-, and 2.89-fold was observed at eight cell (8C, 3 h post fertilization, hpf), morula (M, 6 hpf), and blastula (B, 18 hpf) stages, respectively, after normalization with unfertilized egg (used as a control, set to an arbitrary value of 1). A noticeable increase in *Pl-foxo* mRNA expression was observed at early (EG) and late gastrula (LG), and prism stages (PR) (20, 24 and 36 hpf) (26.33, 25.6-fold, and 19.5-fold respectively), and a subsequent decrease at the pluteus stage (PL, 48 hpf), with respect to prism, up to a still considerable increase of 9.75-fold.

### 3.6. Expression of Pl-foxo Regulatory and Target Genes in PI3K-Inhibited Embryos

FoxO proteins are known to initiate apoptosis, promote cell cycle arrest, and also to be involved in stress resistance. They are regulated by several cellular signaling pathways, such as stress-activated MAPKs (JNK, p38) as well as ERK and PI3K-Akt. It is well acknowledged that PI3K-Akt-mediated phosphorylation of FoxO proteins induces their cytoplasmic translocation and degradation, while the non-phosphorylated forms accumulate in the nucleus to induce the expression of target genes [5]. Previously, we had shown that in the sea urchin *P. lividus*, the inhibition of PI3K activity by a specific inhibitor (LY294002, LY), for 1, 3, 24 and 48h, caused a high increase in *Pl-foxo* mRNA levels [23]. Here, we analyzed the expression of some genes predicted to be related to *Pl-foxo* in PI3K-inhibited embryos after 48h of LY treatment, by real-time qPCR. Apart from the expected upregulation of *Pl-foxo* and *Pl-pi3k*, mRNAs corresponding to *bax*, *Pl-Bi-1*, and *Pl-mnsod* were also all upregulated, albeit with different levels of increase (5.75-, 4.26-, 2.2-, 2.4-, 1.9-fold changes, respectively) (Figure 5B).

### 3.7. Predicted Protein–Protein Interactions for FoxO

**Figure 6:**
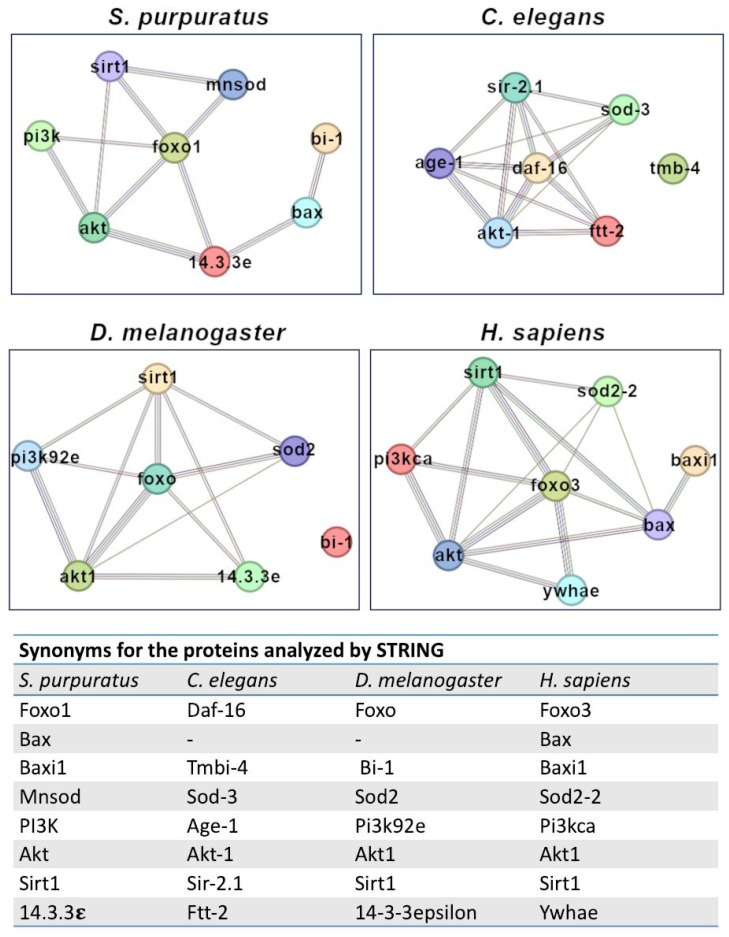
Predicted interaction networks between FoxO proteins and the proteins encoded by the genes analyzed in PI3K-inhibted embryos, i.e., Bax, BI-1, Pi3k, Mnsod, together with Akt, Sirt1 and 14.3.3 e. Protein–protein interaction networks were simulated by the STRING database (confidence interaction score 0.300) in four different organisms, i.e., *S. purpuratus*, *C. elegans*, *D. melanogaster* and *H. sapiens*. Lines linking nodes represent the sources of active interactions, including known (databases, experiments) and predicted (gene neighborhood, fusion, co-occurrence) interactions, as well as text mining (proteins that are frequently mentioned together), co-expression and protein homology. The table shows synonyms for the proteins analyzed by the STRING database for each organism.

In order to predict potential FoxO targets as well as its possible regulators in the sea urchin, we simulated two different protein–protein networks in four different organisms (*S. purpuratus*, *C. elegans*, *D. melanogaster* and *H. sapiens*), using the STRING database (see details in the Section 2). Firstly, we searched for the predicted (physical and/or functional) interactions between FoxO and all the proteins encoded by the genes analyzed by real-time qPCR in the PI3K-inhibited embryos, i.e., Bax, BI-1, Pi3k, and Mnsod, together with Akt, Sirt1 and 14.3.3e (Figure 6).

All these proteins are directly or indirectly involved in apoptosis pathways, and their homologs have been found in the organisms analyzed here, with the exception of Bax, which is not found in *C. elegans* and *D. melanogaster*. Comparing the four protein–protein networks (Figure 6), Sp-foxo1 and its homologs (Ce-daf-16, Dm-foxo and Hs-foxo3) shared protein–protein interactions towards Mnsod, Sirt1, Pi3k, Akt, 14.3.3e, although with different score values. Evidence suggesting a functional link between Sp-foxo1 and these proteins was mainly based on data from putative homologs in other organisms. Interestingly, predictions for Bax interactions were different between *S. purpuratus* and *H. sapiens*, i.e., Sp-bax was predicted to interact with Sp-14.3.3e and Sp-Bi-1, but not with Sp-foxo1, differently from Hs-bax that was predicted to interact with Hs-foxo3, Hs-akt and Hs-BI-1, but not with Hs-ywhae (i.e., 14-3-3) (Figure 6). Ce-tmbi-4 and Dm-Bi-1 (i.e., homologues of Sp-baxi1) showed no predicted interactions with the analyzed proteins.

**Figure 7:**
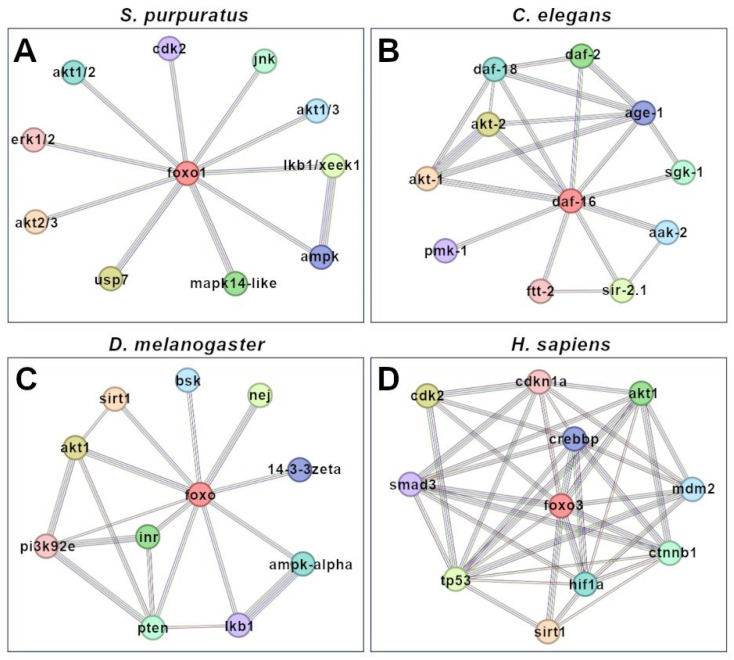
Complete predicted interaction networks of FoxO proteins (Sp-foxo1 and homologs). Protein–protein interaction networks were simulated by searching all predicted interactions using the STRING database (highest confidence interaction score 0.900) in four different organisms, i.e., *S. purpuratus* (**A**), *C. elegans* (**B**), *D. melanogaster* (**C**) and *H. sapiens* (**D**). Lines linking nodes represent the sources of active interactions, including known (databases, experiments) and predicted (gene neighborhood, fusion, co-occurrence) interactions, as well as text mining (proteins that are frequently mentioned together), co-expression and protein homology.

The second type of protein–protein network was simulated by searching for all predicted (physical and functional) interactions for Sp-foxo1 and homologs in the four organisms, setting the highest confidence interaction score (0.900), shown in Figure 7. The predicted interaction between FoxO homologs and Akt1/Akt2 proteins was the only one common among all the four organisms. The predicted interaction between FoxO homologs and Ampk proteins was common among the three invertebrates, while the FoxO1-Mapk14-like predicted interaction was common between *S. purpuratus* and *C. elegans*, and FoxO1-Lkb1 and FoxO1-Mapk10 were common between *S. purpuratus* and *D. melanogaster*. FoxO-Cdk2 was the only predicted interaction common between *S. purpuratus* and *H. sapiens* (Figure 7A,D).

### 3.8. Analysis of Predicted miRNAs Binding to Pl-foxo and Human FoxO3 mRNAs

We identified potential miRNAs that specifically regulate the 3′UTRs of *Pl-foxo* (shown in Figure S1) and *Hs-foxo3* mRNAs, using different bioinformatic tools, mentioned in the Section 2. In particular, to predict miRNAs regulating the 3′UTR of *Pl-foxo* mRNA, it was treated as a human transcript and only miRNAs predicted by both miRanda and PITA were considered positive results. The number of MiRNAs predicted to regulate the 3′ UTR of *Pl-foxo* transcript amounted to 35 (the raw data are reported in Table S1).

The number of experimentally validated miRNAs interacting with the *Hs*-*foxo3* transcripts, found by miRTarBase and selected with a high score, in total amounted to 65. The number of high-confidence predicted miRNAs for the 3′UTR of the main *Hs*-*foxo3* transcript, found by MBS, amounted to 137.

Interestingly, we found few miRNAs that were common to the *Hs*-*foxo3* and *Pl-foxo* transcripts, namely two among the experimentally validated ones (i.e., hsa-miR-665 and hsa-miR-6747-3p), and four among the predicted ones (i.e., hsa-miR-3127-3p, hsa-miR-10394-5p, hsa-miR-6798-5p, and hsa-miR-4758-5p).

Searching for particular miRNAs among the miRTarBase results, we noticed that some interact with the 3’UTR sequence of *Hs*-*foxo3* transcript, i.e., hsa-mir-9-5p, hsa-mir-96, hsa-mir-182 and hsa-mir-29a. All of them are annotated in the miRBASE database as *S. purpuratus* miRNAs named spu-mir-9-5p, spu-mir-96, spu-mr-29a, and spu-mir-182, even if they are not found in the 3’UTR, but in the coding region of *Pl-foxo*. Moreover, it is noteworthy that spu-mir-9-5p shares 72.7% of its identity with hsa-mir-9-5p.

## 4. Discussion

In this study, we report the first evidence for the isolation, characterization and in silico studies of the gene and transcript encoding for the FoxO protein from *P. lividus* sea urchin embryos, referred to as *Pl-foxo*. Only one FoxO gene has been found and annotated in the *P. lividus* genome (in this study), as in *S. purpuratus* [10]. The *Pl-foxo* mRNA showed a high percentage of identity with two sea urchin species, namely *S. purpuratus* and *L. variegatus* (94%), and a lower amount of similarity with the sea stars, as well as with the nematods, insects, chordates and vertebrates analyzed here (shown in Table 1). The two phylogenetic analyses of the *Pl-foxo* protein indicated that this TF is well conserved throughout evolution, sharing both a common ancestor with vertebrates and probably also a similar role. To explain the importance of the gene duplication of TF gene families that encode for similar proteins such as the Fox family, in vertebrates, it is possible to assert what was reviewed by Schmitt-ney [33], who stated that the genic redundancy might be a defense against the accidental loss of a gene and consequently of the gene function, which could cause diseases.

Among members of the human FoxO subfamily, *Pl-foxo* has been shown to be similar to the FoxO3 protein.

The presence of two polyadenylation sites suggested the occurrence of two transcript isoforms, a short and a long one, deriving from alternative splicing. The long isoform originates from using a 5′ splice site near the stop codon and includes all four exons. The short isoform originates from an unspliced exon 2–intron 2 transcript (intron retention type) and stops transcription 250 nt later by using the first polyadenylation site. Both isoforms are supported by cDNA sequences.

Human *foxO* paralogous genes and isoforms have very different gene structures and lengths, containing between two and four exons and ranging from 5 to 125 kb in length [11].

We found at least four predicted regulatory regions of *Pl-foxo* gene expression that can bind putative TFs. The fourth corresponds to the most proximal promoter. Some of the TFs have been annotated in the *S. purpuratus* genome (i.e., *foxf*, *foxg*, *jun*, *fra2*, *six1*, *rfx4*, *maf*, *p3a2*, and *hox11_13b*) and a few of them have also been characterized in *P. lividus*; to name some, *jun* [34], *foxf* and *foxg* [10], and *hox11_13b* [35]. Different members of the KLF (Krüppel-like factor) family of transcription factors are predicted to bind to these regulatory regions. These factors contain zinc-finger domains and are involved in diverse biological processes, including cell proliferation, differentiation, and development [36].

Consistent with the evolutionary conservation of FoxO TFs across the animal kingdom, the sea urchin *Pl-foxo* protein contains all the functional domains characteristic of the family, i.e., the Forkhead box (FH) or the winged-helix DNA-binding domain, conserved during evolution, as well as the neighboring regions. In general, the winged helix motif is the trademark of the Fox protein family and consists of two wings (W1, W2), three α-helices (H1, H2, H3) and three β-sheets (S1, S2, S3), arranged in the following order: H1-S1-H2-H3-S2-W1-S3-W2, as characterized by X-ray crystallography and solution NMR spectroscopy [37]. The structural characterizations of winged helix proteins have highlighted their versatility in DNA binding, which can occur in different modes, and the variety of their biological function, known to be important, for example, in development and aging [37]. *Pl-foxo* has two consecutive α-helices and two consecutive β-sheets; therefore, according to the 3D-model, it could be organized as follows: H1-H2-S1-W1-S2-W2.

The *Pl-foxo* DNA-binding domain is divergent from the vertebrates’ one, especially concerning a deleted part of the *H. sapiens* sequence (from amino acids 109 to 141 of the *Pl-foxo* protein, corresponding to amino acid 40 and 41 in Hs-foxo3 used in this study, and containing one helix and one β-sheet), whereas it is well conserved in all the other organisms considered here, from *D. melanogaster* to vertebrates. Interestingly, within the FH domain, the *Pl-foxo* protein shows two divergent amino acids, even if these are conservative substitutions, i.e., aspartate 110 (D), also present in *S. purpuratus*, *D. melanogaster* and *S. kowaleskii*, instead of the E (glutamic acid) present in the other organisms, and phenylalanine 128 (F), shared only among echinoderms but different from vertebrates and chordates that have Y (tyrosine) (see Figure S4 amino acids in gray, within the most conserved region of the DBD, highlighted with a black cross). This can be due to the different evolutions of the species.

FoxO TFs are mainly regulated through a combination of post-translational modifications (PTMs), including phosphorylation, mono- and polyubiquitination, glycosylation, acetylation, methylation, and nitrosylation [11]. Modifications of FoxO protein conformation occurring through these PTMs create specific sites for binding to protein partners, and affect its subcellular localization and stability, in turn activating/inhibiting the activity of target proteins. The phosphorylation of various aminoacidic residues occurs by several different kinases. As an example, it was demonstrated that the regulation of the FoxO gene is Akt-mediated phosphorylation in response to insulin or growth factors [4]. Phosphorylation by Akt results in the export of FoxO factors from the nucleus to the cytoplasm, thereby inhibiting FoxO-dependent transcription [38]. FoxO proteins are also phosphorylated by other protein kinases, such as JNK or Mst1, under conditions of oxidative stress. This phosphorylation causes the translocation of FoxO from the cytoplasm to the nucleus, the opposite of Akt. JNK-mediated phosphorylation of FoxO4 promotes the expression of anti-oxidative genes, including Mnsod, CAT and GPX, protecting mammalian cells from oxidative damage [39]. Interestingly, such an antioxidant function of FoxO1 is conserved from *C. elegans* to mammals [40]. The prediction of some phosphorylation sites in *Pl-foxo* that are conserved among all the vertebrates and invertebrates analyzed here, namely serine at positions 135, 187, 246, and 255, suggests that these residues are important for the regulation of the protein functions also found in sea urchins.

Protein ubiquitination is an important PTM that regulates the many cellular functions and signaling pathways in eukaryotes, as it can establish the balance between protein degradation and protein longevity [41]. In particular, FoxO proteins can be monoubiquitinated under conditions of oxidative stress, increasing their transcriptional activity, or can be polyubiquitylated and targeted for their degradation [42]. Nevertheless, the *Pl-foxo* analysis conducted by the RUBI software 1.0 did not predict ubiquitylated lysines (K). However, K residues are about 3.6% conserved in Pl-foxo, and their high conservation (ranging from 80 to 100%) (i.e., in residues K132, 142, 165, 176, 179, 202, 203, 204, and 221) was found, compared to the other organisms analyzed in this study (see Clustal W2.1 alignment shown in Figure S3). Studies on the temporal expression of *Sp-foxo1* mRNA, performed measuring transcript abundance, have shown its beginning at the mesenchyme blastula and its high level of increase during gastrulation up to the pluteus stage [10], with a trend very similar to that of the *Pl-foxo* mRNA. The presence of *Pl-foxo* mRNAs in unfertilized eggs is indicative of maternal cytoplasmic storage for later use throughout embryogenesis [43,44]. *Sp-foxo1* was shown to be expressed in mesodermal cells from the mesenchyme blastula stage, i.e., skeletogenic cells and in two distinct secondary mesenchyme populations that likely are blastocoelic and pigment cells [45], and a similar spatial expression of *Pl-foxo* was observed in *P. lividus* at the gastrula stage, although its localization in the archenteron was also observed (Figure S7).

There is currently no experimental evidence of FoxO function in the sea urchin embryo. Nevertheless, on the basis of the known data on the role of its homologs present in other organisms, and on their regulator/target genes, we have been able to address this aspect. In general, FoxO activity appears to be involved in four main processes: apoptosis, cell-cycle arrest, antioxidant response and drug resistance. Each process implies interactions with different genes/proteins, such as FasL and Bax for apoptosis, cyclin D and p27 for cell-cycle arrest, Mnsod and catalase for oxidant response and PI3K/Akt and JNK for drug resistance [5]. Some of these genes have been found in the *P. lividus* genome, and, here, we have selected some of them and investigated their expression correlated with that of *Pl-foxo*. Considering that PI3K is one of the regulators of FoxO function, we have exploited previous studies on PI3K activity in *P. lividus* embryos treated with a specific inhibitor (LY294002, LY), which indeed caused, among other effects, an increase in levels of *Pl-foxo* mRNAs [23]. The confirmed upregulation of *Pl-foxo* and *Pl-pi3k*, as well as that of *Pl-bax*, *Pl-Bi-1* and *Pl-mnsod*, might be interpreted as a response to the stress caused by LY treatment. In particular, the increased expression of *Pl-pi3k* might be an attempt to counteract the inhibition of PI3K enzymatic activity [23], which in turn could be related to the upregulation of its target *Pl-foxo*.

FoxO is known to regulate the pro-apoptotic proteins of the Bcl-2 family, via the direct activation of the *bim* promoter, or the indirect induction of *bax* expression [46], with the latter indeed upregulated in *P. lividus* embryo. However, the absence of the predicted protein–protein interaction between Sp-Foxo1 and Bax proteins in our STRING analyses indicates that to date there is no evidence of direct or indirect interactions between these proteins. The upregulation of *Pl-foxo* and *Pl-bax* mRNAs is the first evidence of a similar potential, although indirect, interaction. On the contrary, such an interaction was predicted for both *D. melanogaster* and *H. sapiens*, excluding *C. elegans* that lacked the *Bax* gene, although the prediction was based on text mining data [47]. Obviously, in all three organisms, the Bax protein was predicted to interact with its inhibitor protein BI-1, on the basis of the association in curated databases and text mining data [48].

Another role of human FoxO3 is the protection from the oxidative damage of cells, carried out by promoting the expression of antioxidant molecules, including Mnsod, CAT and GPX [49]. Interestingly, such an antioxidant function of FoxO3 is conserved from *C. elegans* to vertebrates, thus suggesting a similar role also in sea urchins. The protein–protein interaction between FoxO and Mnsod proteins has been predicted for all four organisms analyzed by STRING.

Aside from the expected prediction of Sp-foxo1 interactions with its regulators PI3k, Akt and 14.3.3e (conserved among homologs from all four organisms), obtained from STRING analyses with a medium score (Figure 6), one interesting, predicted interaction concerns that with the Sirt1 protein. This protein is a deacetylase involved in the control of the cellular response to oxidative stress by regulating FoxO TFs [50]. Sirt1-dependent deacetylation has a dual effect on FoxO3: it increases its ability to induce both cell cycle arrest and resistance to oxidative stress, while it inhibits the FoxO3 ability to induce apoptosis. Sirt1 and FoxO have also been associated with breast cancer progression and metastasis, although the signaling mechanisms underlying their involvement still need to be investigated [51]. The first STRING analyses showed high score values (ranging from 0.918 to 0.999, Figure 6) for the predicted FoxO-Sirt1 interaction in all four organisms analyzed, consistent with conserved functions across invertebrates and vertebrates. However, the second set of STRING analyses, performed by setting the highest confidence interaction score (0.900, Figure 7), unexpectedly did not predict a FoxO-Sirt1 interaction for *S. purpuratus*, rather confirming it in the other three organisms analyzed. Table 4 summarizes the predictions of all proteins interacting with Sp-foxo1 (and its homologs), where common proteins between different organisms are shown in the same line.

Actually, the second STRING network for human FoxO3 is much more complex than that of the three invertebrates analyzed here, having only the predicted FoxO-Akt interaction in common with all of them. The human Akt family contains three members of serine/threonine kinases that are phosphorylated by PI3K. The Akt/PI3K signaling pathway is involved in the regulation of a wide variety of cell processes, i.e., the cell cycle, metabolism, survival, and angiogenesis in normal and malignant cells [52]. Akt is involved in the phosphorylation of FoxO TFs, inducing their binding to 14.3.3 proteins and thus their cytoplasmic localization [38]. This predicted interaction for all the organisms analyzed suggests a conserved role of Akt signaling. In *S. purpuratus*, Akt is encoded by two genes, *Sp-akt-1* and *Sp-akt-2*, which show the highest similarity to the human Akt-3 isoform [53]. The loss of *Sp-akt-2* expression by morpholino antisense oligonucleotides, or the blocking of its function by pharmacological inhibition, impaired cell proliferation in sea urchin embryos [53].

Cdk-2 is a serine/threonine kinase controlling cell cycle progression and is the only other predicted protein interaction with FoxO that *S. purpuratus* and *H. sapiens* have in common (Figure 7). An *Sp-cdk-2* gene has been identified in the *S. purpuratus* genome [54] and its activity in DNA replication has been studied [55].

The activity of FoxO proteins can also be regulated at the post-transcriptional level by several miRNAs [6]. They usually bind to the 3′UTRs of target mRNAs inhibiting their translation or promoting their degradation, and are currently considered very important players in gene regulation. The adopted strategy of using the Hs-foxo3 mRNA sequence was necessary in order to exploit the available bioinformatic tools, which did not allow the use of the sea urchin FoxO sequence in a complete manner as many sea urchin miRNAs do not result as annotated in microRNA databases.

In our study, carried out on the 3′UTRs of *H. sapiens* and *P. lividus*, we found six miRNAs to be common to both the species but annotated only in the *H. sapiens* database (hsa-miR-665, hsa-miR-3127-3p, hsa-miR-6747-3p, hsa-miR-10394-5p, hsa-miR-6798-5p, and hsa-miR-4758-5p). Among those miRNAs, it was interesting to notice that hsa-miR-665 is possibly involved in the regulation of neuroblastoma (NB) progression [56]. In fact, in NB, long non-coding RNA (lncRNA) neuroblastoma highly expressed 1 (NHEG1) negatively regulates the activity of miR-665, which cannot inhibit high mobility group box 1 (HMGB1) expression, causing the aggressive phenotype of neuroblastoma cells. MiR-3127 also seems to play a role in cancer (hepatocellular carcinoma, HCC), as its expression in HCC patients is increased [57]. To our knowledge, the other miRNAs have not been associated with any diseases so far.

The three miRNAs, hsa-mir-9-5p, hsa-mir-96, and hsa-mir-182, whose *S. purpuratus* homologous miRNAs have been annotated in the miRBASE database were found to be highly expressed in HCC liver cancer in a meta-analysis study [58]. To date, their role has not been studied in the sea urchin embryo.

## 5. Conclusions

**Figure 8:**
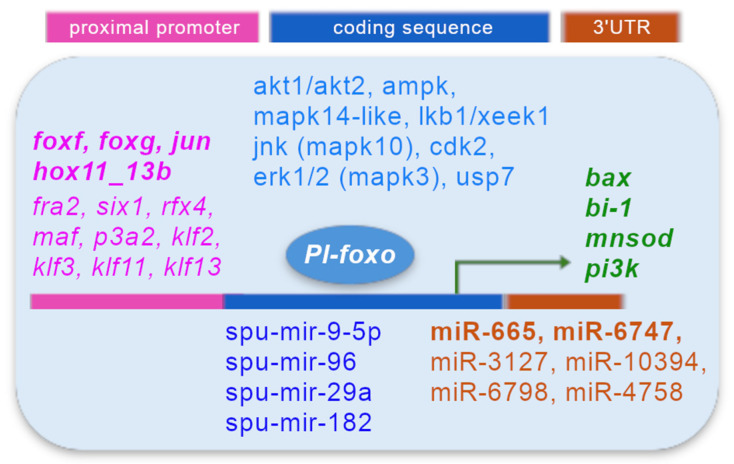
Diagram showing a summary of the experimental and prediction data described in this study. Genes analyzed by qPCR (green); proteins interacting with *Pl-foxo*, predicted by STRING analyses (turquoise); TFs, annotated in the *S. purpuratus* genome, binding the putative regulatory region of the *Pl-foxo* promoter, found by the footprint approach (fuchsia; in bold TFs characterized in *P. lividus*); potential miRNAs for the 3′UTR (orange) and the coding region of *Pl-foxo* (blue), according to database analyses.

In this study, we reported an in-depth characterization of the sea urchin *Pl-foxo* gene and mRNA, predicting its interactions with many regulatory factors, such as proteins and miRNAs, which are conserved between invertebrates and mammals, and suggesting its likely involvement in stress resistance. A diagram is shown in Figure 8, summarizing the *Pl-foxo* TF relationships with the molecules (transcription factors, enzymes, miRNAs) revealed in this study, obtained from our experimental data, such as qPCRs (green) or predictions, such as STRING analyses (turquoise), promoter TF searches (fuchsia), and miRNA database analyses (blue and orange). A comprehensive review of the structure and function of FoxO proteins reports the implications of FoxO in many diseases, and indicates promising potential therapeutic uses of this gene [59].

In conclusion, the presence of the FoxO family of genes in sea urchins and other invertebrates demonstrates a conservative evolution of this gene family. The predicted protein/miRNA interactions that these transcription factors display suggest their important functions in cell signaling pathways and in diseases. Several FoxO functions were reported in the literature but other roles, especially those linked to its dysfunction, remain to be discovered.

## Figures and Tables

**Table 1:** Similarity percentages, with respect to the number of overlapping amino acids, among the *Pl-foxo* protein and homologues from other organisms, with relative Accession numbers and class/phylum of membership.

Organism	Percentage of Similarity/Number of Amino Acids	Accession Number	Protein Name	Class/Phylum
*Paracentrotus lividus*		MT799801.2	Pl-foxo	Echinoids/Echinoderms
*Strongylocentrotus purpuratus*	94/523	XP_030850453	Sp-foxo1	Echinoids/Echinoderms
*Lytechinus variegatus*	94/524	XP_041463504	foxo1-like	Echinoids/Echinoderms
*Acanthaster planci*	61/378	XP_022081651	FOXO3	Asteroidea/Echinoderms
*Asteria rubens*	60/364	XP_033635399	foxo3-like	Asteroidea/Echinoderms
*Holoturia leucospilota*	57/357	KAJ8026986	FOXO	Holothuroidea/Echinoderms
*Crassostrea gigas*	67/210	XP_011414359.1	FOXO	Bivalvia/Molluscs
*Drosophila melanogaster*	62/411	NM_001275628	FOXO	Insects/Arthropods
*Caenorhabditis elegans*	63/328	AF032112.1	DAF16	Chromadorea/Nematoda
*Saccoglossus kowaleskii*	53/347	NP_001158440	FOXO	Enteropneusta/Hemichordates
*Branchiostoma belcheri*	50/316	KAI8520716	FOXO3	Leptocardii/Chordates
*Carcharodon carcharias*	50/312	XP_041068732	FOXO3-like	Fish/Chordates
*Homo sapiens*	50/276	NP_001446.1	FOXO3	Mammals/Chordates

**Table 2:** *Pl-foxo* gene structure and exon–intron boundary sequences (splice sites).

Exon	DNA Range (Sc_218)	Intron (or Upstream)/EXON Seq.	EXON/Intron (or PolyA) Seq.
1	30858789-30859817	tgtggcaggaTTATAAGAGGG	CGGGCTGGAAGgtatgttagt
2	30888244-30889476	tcctgtgcagAACTCTATTCG	TCCATTAAACGgtaagacctg
3	30946237-30946268	ttttgtgcagGTAGTCTATGC	ATCTATCATTGgtaagtagat
4	30970578-30971324	ttccttccagGATATGAATCA	AGTATTTTACAataaaaagaa

**Table 3:** ATAC-seq peaks in the regulatory region of *Pl-foxo* and putative binding TFs.

ATAC-Seq Peak	Transcription Factors
30854289-30855243 Distal promoter	nfyc, shr2, klf13, klf2, sp8, klf11, klf15, cebpg, tll, z145, sox6l, soxf, soxb1, hox8, fos-2, prox, jun, fra2, sia4b, mef2c, dr-1, nk2, zn410, z218, klf3, foxc, foxl2, foxf, foxg, foxd, six1, pax9, pax6, nfe2, atf6b, osr, z116, pou6, nkx62
30856477-30856711	tgif, jun, prox, insm1, soxb2, sox6l, ctl, hnf6, z218, z155
30858032-30858417	rreb1-1, xfin-2, z10, shr2, thr, xbp1, tcp11-1, foxj2, foxl2, foxf, foxg, foxn2, foxd, tf7l2, fos-2, hlf-1, jun, fra2, cebpg, sox6l, z181, z276, sia4b, soxf, arntl, gcnf1, myb, err, z155
30858954-30860032 Proximal promoter	dr-1, six3, z116, z555, klf13, rar, err, ftzf, pou3, z114, zbed, e12, figla, six1, nr1h6b, rfx4, rfx5, xfin-4, maf, klf2, klf3, klf11, p3a2, sp8, glisb, glisc, msand3, pax9, rel-1, irf8, irf4, irf, xfin-2, hxbaa, hox11_13b, insm1, gsc-2, rreb1-1, meis3, tll, osr, ets1b

**Table 4:** Proteins interacting with FoxO found by STRING for the second type of network.

*S. purpuratus*	*C. elegans*	*D. melanogaster*	*H. sapiens*
foxo1	daf-16	foxo	foxo3
akt1/akt2	akt-1/2	akt1	akt1
	sir-2.1	sirt1	sirt1
	daf-2	inr	
	sgk-1		
	daf-18	pten	
ampk	aak-1/aak-2	ampk-alpha (snf1a)	
mapk14-like	pmk-1		
	age-1	pi3k92e	
lkb1/xeek1		lkb1	
		nej	crebbp (ep300)
	ftt-2	14-3-3zeta	
jnk (mapk10)		bsk	
			cdkn1a (p21)
cdk2			cdk2
			smad3
			mdm2
			tp53
			hif1a
			ctnnb1
erik1/2 (mapk3)			
usp7			

## Data Availability

*Pl-foxo* nucleotide sequence data are available in the NCBI databases under the accession number: MT799801.2.

## Supplementary Materials

The following supporting information can be downloaded at: https://www.mdpi.com/article/10.3390/genes15081078/s1, File S1: *P. lividus* database. Figure S1, Nucleotide sequence of *Pl-foxo* contig mRNA. Figure S2, Multiple sequence alignment of *Pl-foxo* ORF nucleotide sequences from the gene annotation, the cDNA clone and the cDNA contig. The deduced amino acid sequence is also shown. Figure S3, Clustal W2.1 alignment of FoxO protein sequences from different organisms. Figure S4, Boxshade alignment of FoxO protein sequences. Black box: high conserved amino acids; gray box: moderately conserved amino acids. Figure S5, Secondary structure of *Pl-foxo* protein predicted by Phyre2 software. Figure S6, Phylogenetic tree of *Pl-foxo* and FoxO proteins of many sea species. Table S1, miRNA analysis of *Pl-foxo* and Hs-foxo3 with different software and intersection data of miRNAs common to the two species. Figure S7, spatial expression of *Pl-foxo* at the gastrula stage.
