# Supplementary material for: Gene, Protein, and in Silico Analyses of FoxO, an Evolutionary Conserved Transcription Factor in the Sea Urchin Paracentrotus lividus
Source: Genes (Basel). 2024 Aug 15;15(8):1078. doi: 10.3390/genes15081078 (PMC11353378; doi:10.3390/genes15081078)
Supplement: Supplementary file 1 [file genes-15-01078-s001.zip › Fig. S1.pdf]

Figure S1a. Nucleotide sequence of the long mRNA isoform of *Pl-foxo* (2757 nt long). 5’ and 3’ UTR are in lowercase. ORF is in uppercase. Start and stop codons are in bold. Polyadenylation signal is underlined.

cactgtagcg ggtacaccaa tctggtcacg attattgtga ttgtgtagtc ccaaactaga 60

gcagacgtat gcattttgca tggcacgaca gccacctgga ctttagtgga tactttttga 120

tgatatcgtc tacctgtgac cagattttgg atttttaaca tcctttggaa acatctttac 180

a**ATG**GTTGAC AACGACCCTG ATTTTGAGCC CCAAGCTCGA CCACGCTCGT GTACATGGCC 240

GTTGCGACGG CCGGACTTTT TGGACTCGAA GCCACAGCAA CCTGGCAATG CCGCCGCCGC 300

GCCTCCGGTA GACCACCCCC ACGGCGCACT CAGTCCTGCC GTCCTAACAG AAGAATCTGT 360

AGATATCAAA CCAATTTTAC CCTTAGAAGG AGGAGAAAAT CGTGAATTGT CAACACCATC 420

ATCTCAAAGA CGGAATGGGT CACGAAGAAA TGCTTGGGGA AATCTGTCAT ACGCAGATTT 480

GATAACGAAA GCTATTCAAA GCGCTCCAGA TCAGCGCTTG ACCCTTTCCC AAATTTACGA 540

CTGGATGGTA AAAAATGTCC CATTTTTCAA GGATAAAGGA GACAGCAATA GTTCAGCGGG 600

CTGGAAGAAC TCTATTCGAC ACAACTTGTC ACTACACAGT CGCTTTGTGC GAGTGCAGAA 660

TGAAGGAACG GGAAAGAGCT CCTGGTGGAT GATCAACCCG GATGCTAAGC CAGGCAAATC 720

ATCAAGGAGA AGAGCATCCA GTATGGACAC CACAAATTCC AAGTTTGAGA GAAAGAGGGG 780

TCGAGTGAAG AAGAAAGTCC TTGAAGAGCG TGCTAAATGG GGTAACACAA GCCCCACACC 840

AAAGCTAGAA GGAGAAGAAG GTGCAAGCCC ACTACCATTC AATCTGGCCA CAACGGATTT 900

CAGATCAAGG GCCAGTTCAA ATGCAAGCAG TTGTGGCCGT CTCTCCCCAA TCATGACCAC 960

ACACCCAGAA ATGGACATGC ATGACAATGA AGTCCCACCC ATGTCTCCAA TTCCCTTCCA 1020

AGATATAGCC CCCTCTCAAG CATACGATAG TCCAGATCCT TACCAGTCCA CAGACCAGCT 1080

AGCTAAACTA GCCAAAGCAA TGACCCTAGA TTCAAGTCTA AGTGTAGAGC CCGCCATCCG 1140

CCACCCACAC AACAACGGTG GATATCTCTT CTCCCCACAA AGCTACAGTG GATCAGATAT 1200

GTCTCCTGTT CATAGTAACA CACAAAGCCC CTATTACTCA CAACAGGGCA CCCCAGCTGT 1260

AAGCCCTCTT GGTCAATGTT CCCCAATGCA GGAATTGCCT CCAAATCAAT ACGGCATGCG 1320

TCAGACCTTC ACCAGCTTGA TGCATGAGAA TAACGATGCC ATCATCCCTC AGGATCCTAT 1380

GTTCTCGCAG ACTGCCGGTT TACGCCAACA GCAGTCACCA CGACCCATGC CAAGCTGTAG 1440

AGAGGAGAGC ATGATCCAGC ACACGTCACC TCATAGGCTG ATGCCTTCTG GGAACCAAGG 1500

CAGCAACCTG GCCATGTTGC TGAACAACGG CCACAACCAG ACCACCAGCC ACCACCACCC 1560

GCTACCTTAT CCTAACGGCG GGACCCCACA CCATATCCCC CATATTCATG CCCATCATCA 1620

GCACCACCAT CATCCCGGCA TAGGGCACCA GGACAGGTTC CCCAGTGACC TGGAGAGTGT 1680

TCAAATTGAC CCCCTCAAAG GATGGAGCGA TCTGGATGTA GAAACAATCC TGAGGAATGA 1740

GCAGGACCTG ACTGAAGGAC CCGATGCCAG CTTTGATAAC ATTGGGACAA TAGGAACCAC 1800

AGCTACAACT ATGGCTGCTC CAAGCTGGGT CCAT**TAA**acg gtagtctatg ctgtgcctac 1860

```
catctatcat tggatatgaa tcaatccaca catactaagg atgatgagaa ttggggataa 1920
aaagagacat cctaccatga ttcctggacc ctatctcgct caaccctgca gtaggggcta 1980
tcaagagata ccttcaggac tcctggaccc tctctcactc aaccctgcag taggggggtac 2040
caatgatctc tcgaaaattc cccctggtgc acaaaaggga aattgttccc ttgcccatta 2100
tatgcctttc gacattttgt tatgaagcgg gtgggatgaa ggaggacctc tagtgtacag 2160
agatggacat agcaccagtt tctgttttct tgttgtaatt catgtcaact tatcattgca 2220
ttcaggctta ggcaggaatc atgacattcc aatcaaatcg tgttgttgtt gttgttatgg 2280
tcaatgtcaa gtgttaaaaa actgataaaa gtctggcatg ctgcctcata gcaagatatc 2340
atggataaaa catcattggg cttgatttaa tcaatatctg aaatataggg attcaagaag 2400
acgaatctcc taatcattac tatcaacagt ctacgatttt aaactagctc atgtggaaga 2460
atcatgttga ctttctttat tacagtattt tacaataaaa agaattatat tagatttttg 2520
tatgaaacaa tagaaaaaaa ttatattaag ctgaagagaa aaacaatggc cttattagta 2580
tatttagaag acaaagtata tgtattacaa atagattatt tcatactcta aactttcaca 2640
gacatagtat atatcagaac tagtttcaag agaaataaaa caatctatgt gtgaagttga 2700
atcatacggg acatatatat gcgatgagct gttcaaggaa acacagaatg catgcat 2757
//
```

Figure S1b. Nucleotide sequence of the short mRNA isoform of Pl-foxo (2089 nt long). 5’ and 3’ UTR are in lowercase. ORF is in uppercase. Start and stop codons are in bold. Polyadenylation signal is underlined. The different sequence of the 3’ UTR is shown in red.

```
cactgtagcg ggtacaccaa tctggtcacg attattgtga ttgtgtagtc ccaaactaga 60
gcagacgtat gcattttgca tggcacgaca gccacctgga ctttagtgga tactttttga 120
tgatatcgtc tacctgtgac cagattttgg atttttaaca tcctttggaa acatctttac 180
aATGGTTGAC AACGACCCTG ATTTTGAGCC CCAAGCTCGA CCACGCTCGT GTACATGGCC 240
GTTGCGACGG CCGGACTTTT TGGACTCGAA GCCACAGCAA CCTGGCAATG CCGCCGCCGC 300
GCCTCCGGTA GACCACCCCC ACGGCGCACT CAGTCCTGCC GTCCTAACAG AAGAATCTGT 360
AGATATCAAA CCAATTTTAC CCTTAGAAGG AGGAGAAAAT CGTGAATTGT CAACACCATC 420
ATCTCAAAGA CGGAATGGGT CACGAAGAAA TGCTTGGGGA AATCTGTCAT ACGCAGATTT 480
GATAACGAAA GCTATTCAAA GCGCTCCAGA TCAGCGCTTG ACCCTTTCCC AAATTTACGA 540
CTGGATGGTA AAAAATGTCC CATTTTTCAA GGATAAAGGA GACAGCAATA GTTCAGCGGG 600
CTGGAAGAAC TCTATTCGAC ACAACTTGTC ACTACACAGT CGCTTTGTGC GAGTGCAGAA 660
TGAAGGAACG GGAAAGAGCT CCTGGTGGAT GATCAACCCG GATGCTAAGC CAGGCAAATC 720
ATCAAGGAGA AGAGCATCCA GTATGGACAC CACAAATTCC AAGTTTGAGA GAAAGAGGGG 780
TCGAGTGAAG AAGAAAGTCC TTGAAGAGCG TGCTAAATGG GGTAACACAA GCCCCACACC 840
AAAGCTAGAA GGAGAAGAAG GTGCAAGCCC ACTACCATTC AATCTGGCCA CAACGGATTT 900
```

CAGATCAAGG GCCAGTTCAA ATGCAAGCAG TTGTGGCCGT CTCTCCCCAA TCATGACCAC 960

ACACCCAGAA ATGGACATGC ATGACAATGA AGTCCCACCC ATGTCTCCAA TTCCCTTCCA 1020

AGATATAGCC CCCTCTCAAG CATACGATAG TCCAGATCCT TACCAGTCCA CAGACCAGCT 1080

AGCTAAACTA GCCAAAGCAA TGACCCTAGA TTCAAGTCTA AGTGTAGAGC CCGCCATCCG 1140

CCACCCACAC AACAACGGTG GATATCTCTT CTCCCCACAA AGCTACAGTG GATCAGATAT 1200

GTCTCCTGTT CATAGTAACA CACAAAGCCC CTATTACTCA CAACAGGGCA CCCCAGCTGT 1260

AAGCCCTCTT GGTCAATGTT CCCCAATGCA GGAATTGCCT CCAAATCAAT ACGGCATGCG 1320

TCAGACCTTC ACCAGCTTGA TGCATGAGAA TAACGATGCC ATCATCCCTC AGGATCCTAT 1380

GTTCTCGCAG ACTGCCGGTT TACGCCAACA GCAGTCACCA CGACCCATGC CAAGCTGTAG 1440

AGAGGAGAGC ATGATCCAGC ACACGTCACC TCATAGGCTG ATGCCTTCTG GGAACCAAGG 1500

CAGCAACCTG GCCATGTTGC TGAACAACGG CCACAACCAG ACCACCAGCC ACCACCACCC 1560

GCTACCTTAT CCTAACGGCG GGACCCCACA CCATATCCCC CATATTCATG CCCATCATCA 1620

GCACCACCAT CATCCCGGCA TAGGGCACCA GGACAGGTTC CCCAGTGACC TGGAGAGTGT 1680

TCAAATTGAC CCCCTCAAAG GATGGAGCGA TCTGGATGTA GAAACAATCC TGAGGAATGA 1740

GCAGGACCTG ACTGAAGGAC CCGATGCCAG CTTTGATAAC ATTGGGACAA TAGGAACCAC 1800

AGCTACAACT ATGGCTGCTC CCAGCTGGGT CCAT**TAA**acg gtaagacctg ctttcatata 1860

actatttact tgtatgatgt ctttgacagt ctacaatgta tgtaaataac gattagttta 1920

gaattccgta attctaatcg atagtgtaaa agattccaaa ttcatattcc catctttgtt 1980

tcaatcacat ttccttataa aagagaagca gtcttgaaaa agcattcatt gtttgacaat 2040

ccaaataaaa cttgttgcag gtcattctac aatggctatc tcccgaacc 2089

//
