# Supplementary material for: Gene, Protein, and in Silico Analyses of FoxO, an Evolutionary Conserved Transcription Factor in the Sea Urchin Paracentrotus lividus
Source: Genes (Basel). 2024 Aug 15;15(8):1078. doi: 10.3390/genes15081078 (PMC11353378; doi:10.3390/genes15081078)
Supplement: Supplementary file 1 [file genes-15-01078-s001.zip › Fig. S2.pdf]

Figure S2. Multiple sequence alignment of Pl-foxo ORF nucleotide sequences from the gene annotation, the cDNA clone and the cDNA contig. The deduced amino acid sequence is also shown. Dots and dashes indicate identities.

```
FoxO_prot_genomic    M  V  D  N  D  P  D  F  E  P  Q  A  R  P  R  S  C  T  W  P  20
Pl_foxo_genomic     ATGgttgacaacgaccctgatttgagccccaagctcgaccacgctcgtgtacatggccg
Pl_foxo_clone       ............................................................
Pl_foxo_contig      ............................................................
FoxO_prot_contig     -  -  -  -  -  -  -  -  -  -  -  -  -  -  -  -  -  -  -  -

FoxO_prot_genomic    L  R  R  P  D  F  L  D  S  K  P  Q  Q  P  G  N  A  A  A  A   40
Pl_foxo_genomic     ttgcgacggccggactttttggactcgaagccacagcaacctggcaatgccgccgccgcg 120
Pl_foxo_clone       ............................................................
Pl_foxo_contig      ............................................................
FoxO_prot_contig     -  -  -  -  -  -  -  -  -  -  -  -  -  -  -  -  -  -  -  -

FoxO_prot_genomic    P  P  V  D  H  P  H  G  A  L  S  P  A  V  L  T  E  E  S  V   60
Pl_foxo_genomic     cctccggtagaccacccccacggcgcactcagtcctgccgtcctaacagaagaatctgta 180
Pl_foxo_clone       ............................................c...............
Pl_foxo_contig      ............................................................
FoxO_prot_contig    -  -  -  -  -  -  -  -  -  -  -  -  -  -  -  -  -  -  -  -

FoxO_prot_genomic    D  I  K  P  I  L  P  L  E  G  G  E  N  R  E  L  S  T  P  S   80
Pl_foxo_genomic     gatatcaaaccaattttacccttagaaggaggagaaaatcgtgaattgtcaacaccatca 240
Pl_foxo_clone       ............................................................
Pl_foxo_contig      ............................................................
FoxO_prot_contig     -  -  -  -  -  -  -  -  -  -  -  -  -  -  -  -  -  -  -  -


FoxO_prot_genomic    S  Q  R  R  N  G  S  R  R  N  A  W  G  N  L  S  Y  A  D  L  100
Pl_foxo_genomic     tctcaaagacggaatgggtcacgaaggaatgcttggggaaatctgtcatacgcagatttg 300
Pl_foxo_clone       ............................................................
Pl_foxo_contig      ..........................a.................................
FoxO_prot_contig     -  -  -  -  -  -  -  -  -  -  -  -  -  -  -  -  -  -  -  -

FoxO_prot_genomic    I  T  K  A  I  Q  S  A  P  D  Q  R  L  T  L  S  Q  I  Y  D  120
Pl_foxo_genomic     ataacgaaagctattcaaagcgctccagatcagcgcttgaccctttcccaaatttacgac 360
Pl_foxo_clone       ............................................................
Pl_foxo_contig      ............................................................
FoxO_prot_contig     -  -  -  -  -  -  -  -  -  -  -  -  -  -  -  -  -  -  -  -

FoxO_prot_genomic    W  M  V  K  N  V  P  F  F  K  D  K  G  D  S  N  S  S  A  G  140
Pl_foxo_genomic     tggatggtaaaaaatgtcccattttttcaaggataaaggagacagcaatagttcagctggt 420
Pl_foxo_clone       ........................................................g..c
Pl_foxo_contig      ........................................................g..c
FoxO_prot_contig     -  -  -  -  -  -  -  -  -  -  -  -  -  -  -  -  -  -  -  -

FoxO_prot_genomic    W  K  N  S  I  R  H  N  L  S  L  H  S  R  F  V  R  V  Q  N  160
Pl_foxo_genomic     tggaagaactctattcgacacaacttgtccctacacagtcgctttgtgcgagtgcagaat 480
Pl_foxo_clone       ............................................................
Pl_foxo_contig      .............................a..............................
FoxO_prot_contig     -  -  -  -  -  -  -  -  -  -  -  -  -  -  -  -  -  -  -  -

FoxO_prot_genomic    E  G  T  G  K  S  S  W  W  M  I  N  P  D  A  K  P  G  K  S  180
Pl_foxo_genomic     gaaggaacaggaaagagctcctggtggatgatcaacccagatgctaagccaggcaaatca 540
Pl_foxo_clone       ............................................................
Pl_foxo_contig      ........g.............................g.....................
FoxO_prot_contig     -  -  -  -  -  -  -  -  -  -  -  -  -  -  -  -  -  -  -  -

FoxO_prot_genomic    S  R  R  R  A  S  S  M  D  T  T  N  S  K  F  E  R  K  R  G  200
Pl_foxo_genomic     tcaagaagaagagcatccagtatggacaccacaaattccaagtttgagagaaagaggggt 600
Pl_foxo_clone       ............................................................
Pl_foxo_contig      .....g......................................................
```

```
FoxO_prot_contig      -  -  -  -  -  -  -  -  -  -  -  -  -  -  -  -  -  -  -  - 

FoxO_prot_genomic     R  V  K  K  K  V  L  E  E  R  A  K  W  G  N  T  S  P  T  P  220
Pl_foxo_genomic      cgagtgaagaagaaagtccttgaagagcgtgctaaatggggtaacacaagccccacacca 660
Pl_foxo_clone        ............................................................
Pl_foxo_contig       ............................................................
FoxO_prot_contig      -  -  -  -  -  -  -  -  -  -  -  -  -  -  -  -  -  -  -  - 

FoxO_prot_genomic     K  L  E  G  E  E  G  A  S  P  L  P  F  N  L  A  T  T  D  F  240
Pl_foxo_genomic      aagctagaaggagaagaaggtgcaagcccactaccattcaatctggccacaacggatttc 720
Pl_foxo_clone        ..............g.............................................
Pl_foxo_contig       ............................................................
FoxO_prot_contig      -  -  -  -  -  -  -  -  -  -  -  -  -  -  -  -  -  -  -  - 

FoxO_prot_genomic     R  S  R  A  S  S  N  A  S  S  C  G  R  L  S  P  I  M  T  T  260
Pl_foxo_genomic      agatcaagggccagttcaaatgcaagcagttgtggccgtctctccccaatcatgaccaca 780
Pl_foxo_clone        .........................................a..................
Pl_foxo_contig       ............................................................
FoxO_prot_contig      -  -  -  -  -  -  -  -  -  -  -  -  -  -  -  -  -  -  -  - 

FoxO_prot_genomic     H  P  E  M  D  M  H  D  N  E  V  P  P  M  S  P  I  P  F  Q  280
Pl_foxo_genomic      cacccagaaatggacatgcatgacaatgaagtcccacccatgtctccaattcccttccaa 840
Pl_foxo_clone        ..t.........................................................
Pl_foxo_contig       ............................................................
FoxO_prot_contig      -  -  -  -  -  -  -  -  -  -  -  -  -  -  -  -  -  -  -  - 

FoxO_prot_genomic     D  I  A  P  S  Q  A  Y  D  S  P  D  P  Y  Q  S  T  D  Q  L  300
Pl_foxo_genomic      gatatagcccccctctcaagcatacgatagtccagatccttaccagtccacagaccagcta 900
Pl_foxo_clone        ............................................................
Pl_foxo_contig       ............................................................
FoxO_prot_contig      -  -  -  -  -  -  -  -  -  -  -  -  -  -  -  -  -  -  -  - 

FoxO_prot_genomic     A  K  L  A  K  A  M  T  L  D  S  S  L  S  V  E  P  A  I  R  320
Pl_foxo_genomic      gctaaactagccaaagcaatgaccctagattcaagtctaagtgtagagcccgccatccgc 960
Pl_foxo_clone        ............................................................
Pl_foxo_contig       ............................................................
FoxO_prot_contig      -  -  -  -  -  -  -  -  -  -  -  -  -  -  -  -  -  -  -  - 

FoxO_prot_genomic     H  P  H  N  N  G  G  Y  L  F  S  P  Q  S  Y  S  G  S  D  M   340
Pl_foxo_genomic      cacccacacaacaacggtggatatctcttctccccacaaagctacagcggatcagatatg 1020
Pl_foxo_clone        .....................................................c..c...
Pl_foxo_contig       ...............................................t............
FoxO_prot_contig      -  -  -  -  -  -  -  -  -  -  -  -  -  -  -  -  -  -  -  - 

FoxO_prot_genomic     S  P  V  H  S  N  T  Q  S  P  Y  Y  S  Q  Q  G  T  P  A  V   360
Pl_foxo_genomic      tctcctgtccatagtaacacacaaagcccctattactcacaacagggcaccccagctgta 1080
Pl_foxo_clone        ..c........c................................................
Pl_foxo_contig       ........t...................................................
FoxO_prot_contig      -  -  -  -  -  -  -  -  -  -  -  -  -  -  -  -  -  -  -  - 

FoxO_prot_genomic     S  P  L  G  Q  C  S  P  M  Q  E  L  P  P  S  Q  Y  N  M  R   380
Pl_foxo_genomic      agccctcttggtcaatgttccccaatgcaggaattgcctccaagtcaatataacatgcgt 1140
Pl_foxo_clone        ...........................................a.......gg.......
Pl_foxo_contig       ...........................................a......cgg.......
FoxO_prot_contig      -  -  -  -  -  -  -  -  -  -  -  -  -  -  N  -  -  G  -  - 

FoxO_prot_genomic     Q  T  F  T  S  L  M  H  E  N  N  D  A  I  I  P  Q  D  P  M   400
Pl_foxo_genomic      cagaccttcaccagcttgatgcatgagaataacgatgccatcatccctcaggatcctatg 1200
Pl_foxo_clone        ............................................................
Pl_foxo_contig       ............................................................
FoxO_prot_contig      -  -  -  -  -  -  -  -  -  -  -  -  -  -  -  -  -  -  -  - 

FoxO_prot_genomic     F  S  Q  T  A  G  L  R  Q  Q  Q  S  P  R  P  M  P  S  C  R   420
Pl_foxo_genomic      ttctcgcagactgccgggttacgccaacagcagtcaccacgacccatgccaagctgtaga 1260
```

```
Pl_foxo_clone        .................t..........................................
Pl_foxo_contig       .................t..........................................
FoxO_prot_contig      -  -  -  -  -  -  -  -  -  -  -  -  -  -  -  -  -  -  -  -

FoxO_prot_genomic     E  E  S  M  I  Q  H  T  S  P  H  R  L  M  P  S  G  N  Q  G   440
Pl_foxo_genomic      gaggagagcatgatccagcacacgtcacctcataggctgatgccttctgggaaccaaggc 1320
Pl_foxo_clone        .......................a....................................
Pl_foxo_contig       ............................................................
FoxO_prot_contig      -  -  -  -  -  -  -  -  -  -  -  -  -  -  -  -  -  -  -  -

FoxO_prot_genomic     S  N  L  A  M  L  L  N  N  G  H  N  Q  T  T  S  H  H  H  P   460
Pl_foxo_genomic      agcaacctggccatgttgctaaacaacggccacaaccagaccaccagccaccaccacccg 1380
Pl_foxo_clone        ............................................................
Pl_foxo_contig       ....................g.......................................
FoxO_prot_contig      -  -  -  -  -  -  -  -  -  -  -  -  -  -  -  -  -  -  -  -

FoxO_prot_genomic     L  P  Y  P  N  G  G  T  P  H  H  I  P  H  I  H  A  H  H  Q   480
Pl_foxo_genomic      ctaccttatcctaacggcgggaccccacaccatatcccccatattcatgcccatcatcag 1440
Pl_foxo_clone        ............................................................
Pl_foxo_contig       ............................................................
FoxO_prot_contig      -  -  -  -  -  -  -  -  -  -  -  -  -  -  -  -  -  -  -  -


FoxO_prot_genomic     H  H  H  H  P  G  I  G  H  Q  D  R  F  P  S  D  L  E  S  V   500
Pl_foxo_genomic      caccaccatcatcccggcatagggcaccaggacaggttccccagtgacctggagagtgtt 1500
Pl_foxo_clone        ............................................................
Pl_foxo_contig       ............................................................
FoxO_prot_contig      -  -  -  -  -  -  -  -  -  -  -  -  -  -  -  -  -  -  -  -

FoxO_prot_genomic     Q  I  D  P  L  K  G  W  S  D  L  D  V  E  T  I  L  R  N  E   520
Pl_foxo_genomic      caaattgacccccctgaaaggatggagcgatctggatgtagaaacaatcctgaggaatgag 1560
Pl_foxo_clone        ..............c.............................................
Pl_foxo_contig       ..............c.............................................
FoxO_prot_contig      -  -  -  -  -  -  -  -  -  -  -  -  -  -  -  -  -  -  -  -

FoxO_prot_genomic     Q  D  L  T  E  G  P  D  A  S  F  D  N  I  G  T  I  G  T  T   540
Pl_foxo_genomic      caggacctgactgaaggacccgatgccagctttgataacattgggacgataggaaccaca 1620
Pl_foxo_clone        ............................................a...............
Pl_foxo_contig       ...............................................a............
FoxO_prot_contig      -  -  -  -  -  -  -  -  -  -  -  -  -  -  -  -  -  -  -  -

FoxO_prot_genomic     A  T  T  M  A  A  P  S  W  V  H  -   551
Pl_foxo_genomic      GctacaactatggctgctcccagctgggtccatTAA 1656
Pl_foxo_clone        ....................................
Pl_foxo_contig       ....................................
FoxO_prot_contig      -  -  -  -  -  -  -  -  -  -  -  -
```
