# Supplementary material for: Gene, Protein, and in Silico Analyses of FoxO, an Evolutionary Conserved Transcription Factor in the Sea Urchin Paracentrotus lividus
Source: Genes (Basel). 2024 Aug 15;15(8):1078. doi: 10.3390/genes15081078 (PMC11353378; doi:10.3390/genes15081078)
Supplement: Supplementary file 1 [file genes-15-01078-s001.zip › Fig. S3.pdf]

Figure S3 clustal W alignment of Pl-foxo with other FoxO proteins.

```
                                                          1            14
PL-FOXO-MT799801.2            ------------------------------------MVDNDPDFEPQARP
SP-FOXO1-XP_030850453         ------------------------------------MVDNDPDFEPQARP
HL-FOXO-KAJ8026986.1          ------------------------------------MDDIDADFEPQDRP
AR-FOXO-XP_033635399.1        ------------------------------------MAEIDPDFEPQSRP
SK-FOXO3-NM_001164968.1       ---------------------------------MADILEIDPDFEPQSRP
HS-FOXO3-NM_001415139.1       --------------------------------------------------
CC-FOXO-XM041212798.1         -----------------------------MEEAVAPHVDIDPDFEPQSRP
BF-FOXO-XP_035685890.1        ------------------------------MDMEDPQLEIDPDFEPQSRP
DM-FOXO-NM_001275628.1        ----------MMDGYAQEWPRLTHTDNGLAMDQLGGDLPLDVGFEPQTRA
Ce-DAF16-AF032112.1           MMEMLVDQGTDASSSASTSTSSVSRFGADTFMNTPDDVMMNDDMEPIPRD

                              15                                      56
PL-FOXO-MT799801.2            RSCTWP-LRRPDFLDSKPQQPGNAAAAPPVDHPHGALSPAVLT-------
SP-FOXO1-XP_030850453         RSCTWP-LRRPDFLDSKPGQPGNAAGAPPVDHAHSALSPAVLA-------
HL-FOXO-KAJ8026986.1          RSCTWPSLRRPEFLDKKPSQAGADQQAQAAQQAQLPEEAQSQA-------
AR-FOXO-XP_033635399.1        RSCTWP-LRRPDFLEKPSQPS--PGEGAPSTGTGAPDEIQDPV-------
SK-FOXO3-NM_001164968.1       RSCTWP-LPRPDFSAQKPSPPPSDQAATPETEIPQEGIEIKQD-------
HS-FOXO3-NM_001415139.1       --------------------------------------------------
CC-FOXO-XM041212798.1         RSCTWP-LPRPEFPVAEGKGEESASHNEPAAATESGGGSGGGGGGVK---
BF-FOXO-XP_035685890.1        RSCTWP-LPRPEVCTNESKTEGSPENEQTTTGEGGGIEPKTEAGTVPG--
DM-FOXO-NM_001275628.1        RSNTWPCPRPENFVEPTDELDSTKASNQQLAPGDSQQAIQNAN-------
Ce-DAF16-AF032112.1           RCNTWP-MRRPQLEPPLNSSPIIHEQIPEEDADLYGSNEQCGQLGGASSN

                                       57                  77  81    87        97
PL-FOXO-MT799801.2            ---------EESVDIKPILPLEGGENRELSTPSSQRRNGSRRNAWGNLSY
SP-FOXO1-XP_030850453         ---------EEPLDIKPVLPLEGGENRELSTPSSQRRNGSRRNAWGNLSY
HL-FOXO-KAJ8026986.1          ---------VASPQITPES------KVDLTTPTTQRKNGSRRNAWGNLSY
AR-FOXO-XP_033635399.1        ---------LSQQVESPDI------KQDLAAVATNRKNCSRRNAWGNLSY
SK-FOXO3-NM_001164968.1       ---------QSTPS---------------------RKNSSRRNAWGNLSY
HS-FOXO3-NM_001415139.1       ----------------------MRLPRSLGHGQVRRGGRGRRRGKGERTG
CC-FOXO-XM041212798.1         --------------AEAKALAATHSPLRLSECSQHRKKSSRRNAWGNLSY
BF-FOXO-XP_035685890.1        ------LAAEPTAPVAAAPTTVDATSLQAELQAPQPKKSSRRNAWGNLSY
DM-FOXO-NM_001275628.1        ---------------------------------AAKKNSSRRNAWGNLSY
Ce-DAF16-AF032112.1           GSTAMLHTPDGSNSHQTSFPSDFRMSESPDDTVSGKKTTTRRNAWGNMSY

                              98                                   135       146
PL-FOXO-MT799801.2            ADLITKAIQSAPDQRLTLSQIYDWMVKNVPFFKDKGDSNSSAGWKN-SIR
SP-FOXO1-XP_030850453         ADLITKAIQSAPDQRLTLSQIYDWMVKNVPFFKDKGDSNSSAGWKN-SIR
HL-FOXO-KAJ8026986.1          ADLITKAIQGAPEQRLTLAQIYEWMVKNVPFFKDKGDSNSSAGWKQNSIR
AR-FOXO-XP_033635399.1        ADLITKAIQSAPEQRLTLSQIYDWMVKNVPFFKDKGDSNSSAGWKN-SIR
SK-FOXO3-NM_001164968.1       ADLITKAIESAPDKRLTLSQIYEWMVKSVPYFKDKGDSNSSAGWKN-SIR
HS-FOXO3-NM_001415139.1       VHLLDSPDSTE---------------------------------EN-SIR
CC-FOXO-XM041212798.1         ADLITKAIESSPEKRLTLSQIYDWMVRHVPYFKDKGDSNSSAGWKN-SIR
BF-FOXO-XP_035685890.1        ADLITKAIQSSPEGRLTLSQIYDWMVRCVPYFRDKGDSNSSAGWKN-SIR
DM-FOXO-NM_001275628.1        ADLITHAIGSATDKRLTLSQIYEWMVQNVPYFKDKGDSNSSAGWKN-SIR
Ce-DAF16-AF032112.1           AELITTAIMASPEKRLTLAQVYEWMVQNVPYFRDKGDSNSSAGWKN-SIR

                              147                                       187   193
PL-FOXO-MT799801.2            HNLSLHSRFVRVQNEGTGKSSWWMINPDAKP---GKSSRRRASSMDTTNS
SP-FOXO1-XP_030850453         HNLSLHSRFVRVQNEGTGKSSWWMINPDAKP---GKSSRRRASSMDTTNS
HL-FOXO-KAJ8026986.1          HNLSLHSRFVRVQNEGTGKSSWWMINPDAKP---GKNSRRRSSSMDTSNA
AR-FOXO-XP_033635399.1        HNLSLHSRFVRVQNEGTGKSSWWMINPDAKP---GKSSRRRASSMDTSNP
SK-FOXO3-NM_001164968.1       HNLSLHSRFVRVQNEGTGKSSWWMINPDATKT--GKSSRRRATSMDTSNK
HS-FOXO3-NM_001415139.1       HNLSLHSRFMRVQNEGTGKSSWWIINPDGGKS--GKAPRRRAVSMDNSNK
CC-FOXO-XM041212798.1         HNLSLHSRFIRVQNEGTGKSSWWMLNPDGGKT--GKSPRRRAVSMDSNSK
BF-FOXO-XP_035685890.1        HNLSLHSRFIRVQNEGTGKSSWWMLNPD-AKG--GKSPRRRASSMDTNNS
DM-FOXO-NM_001275628.1        HNLSLHNRFMRVQNEGTGKSSWWMLNPEAKP---GKSVRRRAASMETSRY
Ce-DAF16-AF032112.1           HNLSLHSRFMRIQNEGAGKSSWVINPDAKPGRNPRRTRERSNTIETTTK
```

```
                           194                     219        227
PL-FOXO-MT799801.2         KFERKRGRVKKKVLEERAKWGNTSPTP----KLEGEEG------------
SP-FOXO1-XP_030850453      KFERKRGRVKKKVLEERAKWGNTSPTP----KLEGEEG------------
HL-FOXO-KAJ8026986.1       KWEKKRGRAKKKAEEKAKNCY-PNSSP----KLEGADD------------
AR-FOXO-XP_033635399.1     KWEKKRGRAKKKVLEEHAKWN-TSPTP----KFEGGEP------------
SK-FOXO3-NM_001164968.1    QFEKKRGRAKKKAAELAAKLAAAGERP----KWSPQTPDGSDTGM-----
HS-FOXO3-NM_001415139.1    YTKSRGRAAKKKAALQTAPESADDSPS-QLSKWPGSP-------------
CC-FOXO-XM041212798.1      YLKSKRGANKKKATLQATQEGNEGSPSSQHTKWSGSP-------------
BF-FOXO-XP_035685890.1     KYEKKRGRAKKKAQEAREAAAAANAGS-PTGKWPGSPQDSKSEGGTPSGD
DM-FOXO-NM_001275628.1     EKRRGRAKKRVEALRQAGVVGLNDATPSPSSSVSEGLDHFPES-------
Ce-DAF16-AF032112.1        AQLEKSRRGAKKRIKERALMGSLHSTLNGNSIAGSIQTISHDLYD-----

                           228                   246         255 259     269
PL-FOXO-MT799801.2         -----ASPLPFNLATTDFRSRASSNASSCG---RLSPIMTTHPEMDMHDN
SP-FOXO1-XP_030850453      -----ASPLPFNLATTDFRSRASSNASSCG---RLSPIMTTHPEIDMHDN
HL-FOXO-KAJ8026986.1       -----QNSLAFSLS-TDFRSRASSNASSCG---RLTPIMAN-ELTDMHDS
AR-FOXO-XP_033635399.1     -----ETSLSLALS-SEFRSRASSNASSCG---RLSPINQMVELTDMHDN
SK-FOXO3-NM_001164968.1    ----VESPLPAFQLSPDFRPRTSSNASSCG---RLSPIMANQELDDMHDN
HS-FOXO3-NM_001415139.1    ----TSRSSDELDAWTDFRSRTNSNASTVSG--RLSPIMASTELDEVQDD
CC-FOXO-XM041212798.1      ----SSHASDEFDAWTDFRSRANSAASTLSG--RLSPIMANSELDELEDD
BF-FOXO-XP_035685890.1     GNLSASTASPLSFNISDFRQRTSSNASSLSG--RLSPIMGPDLDDDNQVP
DM-FOXO-NM_001275628.1     ----PLHSGGGFQLSPDFRQRASSNASSCG---RLSPIRAQDLEPDWGFP
Ce-DAF16-AF032112.1        ---DDSMQGAFDNVPSSFRPRTQSNLSIPGSSSRVSPAIGSDIYDDLEFP

                           270                 290  294             314  317
PL-FOXO-MT799801.2         EVPPMSPIP--FQDIAPSQAYDSPDPYQSTDQLAKLAKAMTLDSSLSVEP
SP-FOXO1-XP_030850453      EVPPMSPIP--FQDLPPSQAYDSPDPYQSTDQLAKLAKAMTLDSSLSVEP
HL-FOXO-KAJ8026986.1       EAPPMSPVPFVDHIGPPHHSFGDSQDSNHTAELTSLAKAMSLNSAMNSPL
AR-FOXO-XP_033635399.1     EAPPMSPG---AYDIGHHTQPYESPDHLHTDQLTSLAQAMSLNASLNGSV
SK-FOXO3-NM_001164968.1    EVPPMSPGPIDWNSTVPVSNYPGAELLRQTTDQLTSALAETMTLNSPDPM
HS-FOXO3-NM_001415139.1    D-APLSPMLYSS-SASLSPSVSKPC-TVELPRLTDMAGTMNLNDGLTENL
CC-FOXO-XM041212798.1      DRTPSSPLLYPSPSNTLSPSVSTRR-TVELPRLADMASTINLNEGLTENL
BF-FOXO-XP_035685890.1     PMSPGWSDFGSNNNLSYG-TSDFLN-QSTDQLTQSLQQTMKLNSPDQLLG
DM-FOXO-NM_001275628.1     VDYQNTTMTQAHAQALEELTGTMADELTLCNQQQQGFSAASGLPSQPPPP
Ce-DAF16-AF032112.1        SWVGESVPAIPSDIVDRTDQMRIDATTHIGGVQIKQESKPIKTEPIAPP

                                                         318       328     335
PL-FOXO-MT799801.2         ------------------------------AIRHPHNNGGYLFS--PQSY
SP-FOXO1-XP_030850453      ------------------------------AIRHTHNNGGYLFS--PQSY
HL-FOXO-KAJ8026986.1       NPDQ---MEQLTVPG---YHVSPQNNGGHYHSSGTSNGNGYIYSPSSSQY
AR-FOXO-XP_033635399.1     SSDLNSSVEQLRVPAPRRRPSPQHTSRGPLFQNGVSNNSNGYMFSPSPS-F
SK-FOXO3-NM_001164968.1    TIDQLTLSPQPQIQP------AQSPSPGLPAVYTNQNGSTFTLSTQAPTF
HS-FOXO3-NM_001415139.1    MDDLLDNITLPPSQP-------------SPTGGLMQRSSSFPYTTKGSGL
CC-FOXO-XM041212798.1      LEDLQDNYNMSPSQQ-------------IPSGCLRQRSSSFSFGSKCSTR
BF-FOXO-XP_035685890.1     DSGLSGMSSMDSVGS-------------LGLGGFDSQPDPFMRRCHSVGS
DM-FOXO-NM_001275628.1     PYQPPQHQQAQQQQQ---------QQSPYALNGPASGYNTLQPQSQCLLH
Ce-DAF16-AF032112.1        ---------------------------------SYHELNSVRGSCAQNPL

                           336                                            376
PL-FOXO-MT799801.2         SGSDMSPVHSNTQSPYYS---------QQGTPAVSPLGQCSPMQELPPNQ
SP-FOXO1-XP_030850453      SGSDMSPVHS--NSPYYS---------QQGTPAVSPLGQCSPMQELPPSQ
HL-FOXO-KAJ8026986.1       SGSELSPAQNGVQSPFSSYS-------QPNTPVMSPINQPQPQQQQQCSP
AR-FOXO-XP_033635399.1     SGSDISPVHSNVQSPAYSPY-------GQNS-------AMSPMAQQRCSP
SK-FOXO3-NM_001164968.1    GTQNVTLANTSLFLPYNTQSGLCMTQAGQTSPSSLGMISENPSPPQLSMS
HS-FOXO3-NM_001415139.1    GSPTSSFNSTVFGPSSLNSL------------------RQSPMQTIQENK
CC-FOXO-XM041212798.1      GSQTSTYSATMYSQPPMTML------------------RHSPMQTIQENK
BF-FOXO-XP_035685890.1     RAQLPSPRRQNTGYSTPPPS------------------FKSPFSPVQVPQ
DM-FOXO-NM_001275628.1     RSLNCSCMHNARDGLSPNSVT----------------TTMSPAYPNSEPS
Ce-DAF16-AF032112.1        LRNPIVPSTNFKPMPLPGAYG-----------------------------
```

```
                        377                                               406
PL-FOXO-MT799801.2      YG--------------------MRQTFTSLMHENNDAIIPQDPMFSQTAG
SP-FOXO1-XP_030850453   YG--------------------MQRSFTNLIHEN-ESIIPQDPMFAQSAV
HL-FOXO-KAJ8026986.1    INGVSDLNP--------QYIQQPPGLLSQSANILSQDPIQNRLNVPADSV
AR-FOXO-XP_033635399.1  SMSVTDMSQPFTMNVPQQQQQQTPSAFTMMPHQDPSVILSKRDPMLSHST
SK-FOXO3-NM_001164968.1 NIQQVSQTPLNEAMISQNDPILPSDLIISQQDPMLSQISGNDLMLQSDPM
HS-FOXO3-NM_001415139.1 PATFSSMS------HYGNQTLQDLLTSDSLSHSDVMMTQSDPLMSQASTA
CC-FOXO-XM041212798.1   QVTFSTIN------HYGNRMLQDLLTPESLRHKEVMMTQTDPLMPQANTV
BF-FOXO-XP_035685890.1  PAHSPNQQQ--GSLPSVNAPQQDPTQFSLQQLSDVMLTQTDPILTSGDPV
DM-FOXO-NM_001275628.1  SDSLNTYSNVVLDGPADTAALMVQQQQQQQQQQQLSASLEDNNCASTLIG
Ce-DAF16-AF032112.1     --------------NYQNGGITPINWLSTSNSSPLPGIQSCGIVAAQHTV

                        407           418  423                            427
PL-FOXO-MT799801.2      LRQQ----QSPRPMPSCREESM-------------------------IQH
SP-FOXO1-XP_030850453   LRQQ----QSPRPMPSCREESM-------------------------NQH
HL-FOXO-KAJ8026986.1    MMQQAVHNQS--MLSSCREQSN-------------------------MVH
AR-FOXO-XP_033635399.1  LGPGSMMHQRPTNLPTCRVQTS-------------------------ISQ
SK-FOXO3-NM_001164968.1 LSMAQEQKYVPSRLNFNIPSSVNLTQPN-----------------VMTGF
HS-FOXO3-NM_001415139.1 VSAQN---SRRNVMLRNDPMMS-----------------------FAAQP
CC-FOXO-XM041212798.1   VASQN---QRQMVMCSDPAISP-----------------------FNAQS
BF-FOXO-XP_035685890.1  MSSGDPLISQGNLMLRQDPMMSGCPDQGSQLSSLLQGTGMSYAFRQQQQQ
DM-FOXO-NM_001275628.1  QCLEVLNNEAQPIDEFNLENFPVG----------------------NLEC
Ce-DAF16-AF032112.1     ASSSALPIDLENLTLP----------------------------------

                        428                                               474
PL-FOXO-MT799801.2      TSPHRLMPS-GNQGSNLAMLLNNGHN--QTTSHHHPLPYPNGGTPHHIPH
SP-FOXO1-XP_030850453   TSPHRLMPSSMNQGSNLAMLLNNGHNTHTTSHHHHPQPYPNGGTPHHVHP
HL-FOXO-KAJ8026986.1    R--QRFVSPGQHQQVNQSSLALALGMS---QQGFTPASLQMSRNIQAYQQ
AR-FOXO-XP_033635399.1  GRSLQGFSPQTSPSNPLSGLLLPNAVT---ASQTNHMSSPTHVSLNAHYA
SK-FOXO3-NM_001164968.1 GNNLQPFQQQQQQQQHSRLLAQLIGGNALQQQLQQQQQQQQQQQQQQQQQ
HS-FOXO3-NM_001415139.1 NQGSLVNQNLLHHQHQTQGALGGSRALSNSVSNMGLSESSSLGSAKHQQQ
CC-FOXO-XM041212798.1   PRLMNSNPFHHPSTAQQNSAVNNGALSNPIGLMHMHSDAGNINSVAHHLQ
BF-FOXO-XP_035685890.1  QLQQQQQQQQQPQQQPQQQNYNLGCALNQMLGGNMGIMAGQLAPTQQQQQ
DM-FOXO-NM_001275628.1  NVEELLQQEMSYGGLLDINIPLATVNTNLVNSSSGPLSISNISNLSNISS
Ce-DAF16-AF032112.1     -------------------------------DQPLMDTMDVDALIRHELS

                        475                 495                         505
PL-FOXO-MT799801.2      IHAH-HQHHHHPGIGHQDRFPSDLES------------------VQIDPL
SP-FOXO1-XP_030850453   HHIHPHIHHHHPGMVHVDRFPSDLES------------------VQIDPL
HL-FOXO-KAJ8026986.1    QSTQQNLQSQLVPN-----GPIPNDL------------------VSIEVE
AR-FOXO-XP_033635399.1  QSMNQLLQSSPMTTNTSDRVPTDLQS------------------LNVDMF
SK-FOXO3-NM_001164968.1 QQQQQMLSNQRTNFSMLQSMNMNMNMPGLATDQLTNMHDKFPSDLELDMF
HS-FOXO3-NM_001415139.1 ---SPVSQSMQTLSDSLSGSSLYSTS----ANLPVMGHEKFPSDLDLDMF
CC-FOXO-XM041212798.1   NQLHSLASHGMQMEASDSRLSSCPGG----INISTMSQDKFPTDLDLDMF
BF-FOXO-XP_035685890.1  TPLPQISTFGMMHQQSPASLHQQAMANSQANQLLSMHQEKFPSDLELDMF
DM-FOXO-NM_001275628.1  NSGSSLSLNQLQAQLQQQQQQQQAQQ------------------QQQAQQ
Ce-DAF16-AF032112.1     QAGGQHIHFDL---------------------------------------

                        506                                               543
PL-FOXO-MT799801.2      KGWSDLDVETILRNE-----------QDLTEG-PDASFDNIGTIGTTATT
SP-FOXO1-XP_030850453   KGWSDLDVETILRNE-----------QDLTEG-PDASFDNIGTIGTTATT
HL-FOXO-KAJ8026986.1    P-EFDIDMDSFIRNE-----------VNLGDGFNNVNFDNINTGNPTSTA
AR-FOXO-XP_033635399.1  K-DMHCDIESVIHDE-----------LKMDDGNLDFNFD-----GPINNQ
SK-FOXO3-NM_001164968.1 NGGLECDVDSIIRNEPDITETAALDFNNLEGNTSSTMGMNLTFSTSSATS
HS-FOXO3-NM_001415139.1 NGSLECDMESIIRSELMDAD--GLDFNFDSLISTQNVVGLNVGNFTGAKQ
CC-FOXO-XM041212798.1   HGSLECDVESIILNEFMDSE--ELDFNFDCAMPTQSVG-INMATLPTAPQ
BF-FOXO-XP_035685890.1  SGGLECDMDSIINTELMEDG--GLEFNFQQDPNNQMGSCAPTSAGMTQSM
DM-FOXO-NM_001275628.1  QQQHQQHQQQLLLNNNNNSS----SSLELATQTATTNLNARVQYSQPSVV
Ce-DAF16-AF032112.1     --------------------------------------------------
```

```
                              544   551
PL-FOXO-MT799801.2            MAAPSWVH-----------
SP-FOXO1-XP_030850453         MAAPSWVH-----------
HL-FOXO-KAJ8026986.1          NIGTNWVH-----------
AR-FOXO-XP_033635399.1        TVAPNWVH-----------
SK-FOXO3-NM_001164968.1       SSTQSWVH-----------
HS-FOXO3-NM_001415139.1       ASSQSWVPG----------
CC-FOXO-XM041212798.1         TTNQSWVPG----------
BF-FOXO-XP_035685890.1        TMSQTQTNAGRLGYRETVI
DM-FOXO-NM_001275628.1        TSPPSWVH-----------
Ce-DAF16-AF032112.1           -------------------
```
