# Supplementary material for: Gene, Protein, and in Silico Analyses of FoxO, an Evolutionary Conserved Transcription Factor in the Sea Urchin Paracentrotus lividus
Source: Genes (Basel). 2024 Aug 15;15(8):1078. doi: 10.3390/genes15081078 (PMC11353378; doi:10.3390/genes15081078)
Supplement: Supplementary file 1 [file genes-15-01078-s001.zip › FIG. S4.pdf]

```
                              1   .........10........20........30........40........50
PL-FOXO-MT799801.2            1   ------------------------------------MVDNDPDFEPQARP
SP-FOXO1-XP_030850453         1   ------------------------------------MVDNDPDFEPQARP
HL-FOXO-KAJ8026986.1          1   ------------------------------------MDDIDADFEPQDRP
AR-FOXO-XP_033635399.1        1   ------------------------------------MAEIDPDFEPQSRP
SK-FOXO3-NM_001164968.1       1   ---------------------------------MADILEIDPDFEPQSRP
HS-FOXO3-NM_001415139.1       1   --------------------------------------------------
CC-FOXO-XM041212798.1         1   -----------------------------MEEAVAPHVDIDPDFEPQSRP
BF-FOXO-XP_035685890.1        1   ------------------------------MDMEDPQLEIDPDFEPQSRP
DM-FOXO-NM_001275628.1        1   ----------MMDGYAQEWPRLTHTDNGLAMDQLGGDLPLDVGFEPQTRA
Ce-DAF16-AF032112.1           1   MMEMLVDQGTDASSSASTSTSSVSRFGADTFMNTPDDVMMNDDMEPIPRD

                             51   .........60........70........80........90.......100
PL-FOXO-MT799801.2           15   RSCTWP-LRRPDFLDSKPQQPGNAAAAPPVDHPHGALSPAVLT-------
SP-FOXO1-XP_030850453        15   RSCTWP-LRRPDFLDSKPGQPGNAAGAPPVDHAHSALSPAVLA-------
HL-FOXO-KAJ8026986.1         15   RSCTWPSLRRPDFLDKKPSQAGADQQAQAAQQAQLPEEAQSQA-------
AR-FOXO-XP_033635399.1       15   RSCTWP-LRRPEFLEKPSQPS--PGEGAPSTGTGAPDEIQDPV-------
SK-FOXO3-NM_001164968.1      18   RSCTWP-LPRPDFSAQKPSPPPSDQAATPETEIPQEGIEIKQD-------
HS-FOXO3-NM_001415139.1       1   --------------------------------------------------
CC-FOXO-XM041212798.1        22   RSCTWP-LPRPEFPVAEGKGEESASHNEPAAATESGGGSGGGGGGVK---
BF-FOXO-XP_035685890.1       21   RSCTWP-LPRPEVCTNESKTEGSPENEQTTTGEGGIEPKTEAGTVPG--
DM-FOXO-NM_001275628.1       41   RSNTWPCPRPENFVEPTDELDSTKASNQQLAPGDSQQAIQNAN-------
Ce-DAF16-AF032112.1          51   RCNTWP-MRRPQLEPPLNSSPIIHEQIPEEDADLYGSNEQCGQLGGASSN

                            101   .......110.......120.......130.......140.......150
PL-FOXO-MT799801.2           57   ---------EESVDIKPILPLEGGENRELSTPSSQRRNGSRRNAWGNLSY
SP-FOXO1-XP_030850453        57   ---------EEPLDIKPVLPLEGGENRELSTPSSQRRNGSRRNAWGNLSY
HL-FOXO-KAJ8026986.1         58   ---------VASPQITPES------KVDLTTPTTQRKNGSRRNAWGNLSY
AR-FOXO-XP_033635399.1       55   ---------LSQQVESPDI------KQDLAAVATNRKNCSRRNAWGNLSY
SK-FOXO3-NM_001164968.1      60   ---------QSTPS---------------------RKNSSRRNAWGNLSY
HS-FOXO3-NM_001415139.1       1   ----------------------MRLPRSLGHGQVRRGGRGRRRGKGERTG
CC-FOXO-XM041212798.1        68   --------------AEAKALAATHSPLRLSECSQHRKKSSRRNAWGNLSY
BF-FOXO-XP_035685890.1       68   ------LAAEPTAPVAAAPTTVDATSLQAELQAPQPKKSSRRNAWGNLSY
DM-FOXO-NM_001275628.1       84   ---------------------------------AAKKNSSRRNAWGNLSY
Ce-DAF16-AF032112.1         100   GSTAMLHTPDGSNSHQTSFPSDFRMSESPDDTVSGKKTTTRRNAWGNMSY

                                              X                 X
                            151   .......160.......170.......180.......190.......200
PL-FOXO-MT799801.2           98   ADLITKAIQSAPDQRLTLSQIYDWMVKNVPFFKDKGDSNSSAGWKN-SIR
SP-FOXO1-XP_030850453        98   ADLITKAIQSAPDQRLTLSQIYDWMVKNVPFFKDKGDSNSSAGWKN-SIR
HL-FOXO-KAJ8026986.1         93   ADLITKAIQGAPEQRLTLAQIYEWMVKNVPFFKDKGDSNSSAGWKQNSIR
AR-FOXO-XP_033635399.1       90   ADLITKAIQSAPEQRLTLSQIYDWMVKNVPFFKDKGDSNSSAGWKN-SIR
SK-FOXO3-NM_001164968.1      80   ADLITKAIESAPDKRLTLSQIYEWMVKSVPYFKDKGDSNSSAGWKN-SIR
HS-FOXO3-NM_001415139.1      29   VHLLDSPDSTE---------------------------------EN-SIR
CC-FOXO-XM041212798.1       104   ADLITKAIESSPEKRLTLSQIYDWMVRHVPYFKDKGDSNSSAGWKN-SIR
BF-FOXO-XP_035685890.1      112   ADLITKAIQSSPEGRLTLSQIYDWMVRCVPYFRDKGDSNSSAGWKN-SIR
DM-FOXO-NM_001275628.1      101   ADLITHAIGSATDKRLTLSQIYEWMVQNVPYFKDKGDSNSSAGWKN-SIR
Ce-DAF16-AF032112.1         150   AELITTAIMASPEKRLTLAQVYEWMVQNVPYFRDKGDSNSSAGWKN-SIR

                            201   .......210.......220.......230.......240.......250
PL-FOXO-MT799801.2          147   HNLSLHSRFVRVQNEGTGKSSWWMINPDAKP---GKSSRRRASSMDTTNS
SP-FOXO1-XP_030850453       147   HNLSLHSRFVRVQNEGTGKSSWWMINPDAKP---GKSSRRRASSMDTTNS
HL-FOXO-KAJ8026986.1        143   HNLSLHSRFVRVQNEGTGKSSWWMINPDAKP---GKNSRRRSSSMDTSNA
AR-FOXO-XP_033635399.1      139   HNLSLHSRFVRVQNEGTGKSSWWMINPDAKP---GKSSRRRASSMDTSNP
SK-FOXO3-NM_001164968.1     129   HNLSLHSRFVRVQNEGTGKSSWWMINPDATKT--GKSSRRRATSMDTSNK
HS-FOXO3-NM_001415139.1      45   HNLSLHSRFMRVQNEGTGKSSWWIINPDGGKS--GKAPRRRAVSMDNSNK
CC-FOXO-XM041212798.1       153   HNLSLHSRFIRVQNEGTGKSSWWMLNPDGGKT--GKSPRRRAVSMDSNSK
BF-FOXO-XP_035685890.1      161   HNLSLHSRFIRVQNEGTGKSSWWMLNPD-AKG--GKSPRRRASSMDTNNS
DM-FOXO-NM_001275628.1      150   HNLSLHNRFMRVQNEGTGKSSWWMLNPEAKP---GKSVRRRAASMETSRY
Ce-DAF16-AF032112.1         199   HNLSLHSRFMRIQNEGAGKSSWWVINPDAKPGRNPRRTRERSNTIETTTK

                            251   .......260.......270.......280.......290.......300
PL-FOXO-MT799801.2          194   KFERKRGRVKKKVLEERAKWGNTSPTP----KLEGEEG------------
SP-FOXO1-XP_030850453       194   KFERKRGRVKKKVLEERAKWGNTSPTP----KLEGEEG------------
HL-FOXO-KAJ8026986.1        190   KWEKKRGRAKKKAEEKAKNCY-PNSSP----KLEGADD------------
AR-FOXO-XP_033635399.1      186   KWEKKRGRAKKKVLEEHAKWN-TSPTP----KFEGGEP------------
SK-FOXO3-NM_001164968.1     177   QFEKKRGRAKKKAAELAAKLAAAGERP----KWSPQTPDGSDTGM-----
HS-FOXO3-NM_001415139.1      93   YTKSRGRAAKKKAALQTAPESADDSPS-QLSKWPGSP-------------
CC-FOXO-XM041212798.1       201   YLKSKRGANKKKATLQATQEGNEGSPSSQHTKWSGSP-------------
BF-FOXO-XP_035685890.1      208   KYEKKRGRAKKKAQEAREAAAAANAGS-PTGKWPGSPQDSKSEGGTPSGD
DM-FOXO-NM_001275628.1      197   EKRRGRAKKRVEALRQAGVVGLNDATPSPSSSVSEGLDHFPES-------
Ce-DAF16-AF032112.1         249   AQLEKSRRGAKKRIKERALMGSLHSTLNGNSIAGSIQTISHDLYD-----
```

```
                             301 .......310.......320.......330.......340.......350
PL-FOXO-MT799801.2           228 -----ASPLPFNLATTDFRSRASSNASSCG---RLSPIMTTHPEMDMHDN
SP-FOXO1-XP_030850453        228 -----ASPLPFNLATTDFRSRASSNASSCG---RLSPIMTTHPEIDMHDN
HL-FOXO-KAJ8026986.1         223 -----QNSLAFSLS-TDFRSRASSNASSCG---RLTPIMAN-ELTDMHDS
AR-FOXO-XP_033635399.1       219 -----ETSLSLALS-SEFRSRASSNASSCG---RLSPINQMVELTDMHDN
SK-FOXO3-NM_001164968.1      218 ----VESPLPAFQLSPDFRPRTSSNASSCG---RLSPIMANQELDDMHDN
HS-FOXO3-NM_001415139.1      129 ----TSRSSDELDAWTDFRSRTNSNASTVSG--RLSPIMASTELDEVQDD
CC-FOXO-XM041212798.1        238 ----SSHASDEFDAWTDFRSRANSAASTLSG--RLSPIMANSELDELEDD
BF-FOXO-XP_035685890.1       257 GNLSASTASPLSFNISDFRQRTSSNASSLSG--RLSPIMGPDLDDDNQVP
DM-FOXO-NM_001275628.1       240 ----PLHSGGGFQLSPDFRQRASSNASSCG---RLSPIRAQDLEPDWGFP
Ce-DAF16-AF032112.1          294 ---DDSMQGAFDNVPSSFRPRTQSNLSIPGSSSRVSPAIGSDIYDDLEFP

                             351 .......360.......370.......380.......390.......400
PL-FOXO-MT799801.2           270 EVPPMSPIP--FQDIAPSQAYDSPDPYQSTDQLAKLAKAMTLDSSLSVEP
SP-FOXO1-XP_030850453        270 EVPPMSPIP--FQDLPPSQAYDSPDPYQSTDQLAKLAKAMTLDSSLSVEP
HL-FOXO-KAJ8026986.1         263 EAPPMSPVPFVDHIGPPHHSFGDSQDSNHTAELTSLAKAMSLNSAMNSPL
AR-FOXO-XP_033635399.1       260 EAPPMSPG---AYDIGHHTQPYESPDHLHTDQLTSLAQAMSLNASLNGSV
SK-FOXO3-NM_001164968.1      261 EVPPMSPGPIDWNSTVPVSNYPGAELLRQTTDQLTSALAETMTLNSPDPM
HS-FOXO3-NM_001415139.1      173 D-APLSPMLYSS-SASLSPSVSKPC-TVELPRLTDMAGTMNLNDGLTENL
CC-FOXO-XM041212798.1        282 DRTPSSPLLYPSPSNTLSPSVSTRR-TVELPRLADMASTINLNEGLTENL
BF-FOXO-XP_035685890.1       305 PMSPGWSDFGSNNNLSYG-TSDFLN-QSTDQLTQSLQQTMKLNSPDQLLG
DM-FOXO-NM_001275628.1       283 VDYQNTTMTQAHAQALEELTGTMADELTLCNQQQQGFSAASGLPSQPPPP
Ce-DAF16-AF032112.1          341 SWVGESVPAIPSDIVDRTDQMRIDATTHIGGVQIKQESKPIKTEPIAPPP

                             401 .......410.......420.......430.......440.......450
PL-FOXO-MT799801.2           318 ------------------------------AIRHPHNNGGYLFS--PQSY
SP-FOXO1-XP_030850453        318 ------------------------------AIRHTHNNGGYLFS--PQSY
HL-FOXO-KAJ8026986.1         313 NPDQ---MEQLTVPG---YHVSPQNNGGHYHSSGTSNGNGYIYSPSSSQY
AR-FOXO-XP_033635399.1       307 SSDLNSSVEQLRVPAPRRPSPQHTSRGPLFQNGVSNNSNGYMFSPSPS-F
SK-FOXO3-NM_001164968.1      311 TIDQLTLSPQPQIQP------AQSPSPGLPAVYTNQNGSTFTLSTQAPTF
HS-FOXO3-NM_001415139.1      220 MDDLLDNITLPPSQP-------------SPTGGLMQRSSSFPYTTKGSGL
CC-FOXO-XM041212798.1        331 LEDLQDNYNMSPSQQ-------------IPSGCLRQRSSSFSFGSKCSTR
BF-FOXO-XP_035685890.1       353 DSGLSGMSSMDSVGS-------------LGLGGFDSQPDPFMRRCHSVGS
DM-FOXO-NM_001275628.1       333 PYQPPQHQQAQQQQQ---------QQSPYALNGPASGYNTLQPQSQCLLH
Ce-DAF16-AF032112.1          391 ---------------------------------SYHELNSVRGSCAQNPL

                             451 .......460.......470.......480.......490.......500
PL-FOXO-MT799801.2           336 SGSDMSPVHSNTQSPYYS---------QQGTPAVSPLGQCSPMQELPPNQ
SP-FOXO1-XP_030850453        336 SGSDMSPVHS--NSPYYS---------QQGTPAVSPLGQCSPMQELPPSQ
HL-FOXO-KAJ8026986.1         357 SGSELSPAQNGVQSPFSSYS-------QPNTPVMSPINQPQPQQQQQCSP
AR-FOXO-XP_033635399.1       356 SGSDISPVHSNVQSPAYSPY-------GQNS-------AMSPMAQQRCSP
SK-FOXO3-NM_001164968.1      355 GTQNVTLANTSLFLPYNTQSGLCMTQAGQTSPSSLGMISENPSPPQLSMS
HS-FOXO3-NM_001415139.1      257 GSPTSSFNSTVFGPSSLNSL------------------RQSPMQTIQENK
CC-FOXO-XM041212798.1        368 GSQTSTYSATMYSQPPMTML------------------RHSPMQTIQENK
BF-FOXO-XP_035685890.1       390 RAQLPSPRRQNTGYSTPPPS------------------FKSPFSPVQVPQ
DM-FOXO-NM_001275628.1       374 RSLNCSCMHNARDGLSPNSVT----------------TTMSPAYPNSEPS
Ce-DAF16-AF032112.1          408 LRNPIVPSTNFKPMPLPGAYG-----------------------------

                             501 .......510.......520.......530.......540.......550
PL-FOXO-MT799801.2           377 YG--------------------MRQTFTSLMHENNDAIIPQDPMFSQTAG
SP-FOXO1-XP_030850453        375 YG--------------------MQRSFTNLIHEN-ESIIPQDPMFAQSAV
HL-FOXO-KAJ8026986.1         400 INGVSDLNP--------QYIQQPPGLLSQSANILSQDPIQNRLNVPADSV
AR-FOXO-XP_033635399.1       392 SMSVTDMSQPFTMNVPQQQQQQTPSAFTMMPHQDPSVILSKRDPMLSHST
SK-FOXO3-NM_001164968.1      405 NIQQVSQTPLNEAMISQNDPILPSDLIISQQDPMLSQISGNDLMLQSDPM
HS-FOXO3-NM_001415139.1      289 PATFSSMS------HYGNQTLQDLLTSDSLSHSDVMMTQSDPLMSQASTA
CC-FOXO-XM041212798.1        400 QVTFSTIN------HYGNRMLQDLLTPESLRHKEVMMTQTDPLMPQANTV
BF-FOXO-XP_035685890.1       422 PAHSPNQQQ--GSLPSVNAPQQDPTQFSLQQLSDVMLTQTDPILTSGDPV
DM-FOXO-NM_001275628.1       408 SDSLNTYSNVVLDGPADTAALMVQQQQQQQQQQQLSASLEDNNCASTLIG
Ce-DAF16-AF032112.1          429 --------------NYQNGGITPINWLSTSNSSPLPGIQSCGIVAAQHTV

                             551 .......560.......570.......580.......590.......600
PL-FOXO-MT799801.2           407 LRQQ----QSPRPMPSCREESM-------------------------IQH
SP-FOXO1-XP_030850453        404 LRQQ----QSPRPMPSCREESM-------------------------NQH
HL-FOXO-KAJ8026986.1         442 MMQQAVHNQS--MLSSCREQSN-------------------------MVH
AR-FOXO-XP_033635399.1       442 LGPGSMMHQRPTNLPTCRVQTS-------------------------ISQ
SK-FOXO3-NM_001164968.1      455 LSMAQEQKYVPSRLNFNIPSSVNLTQPN-----------------VMTGF
HS-FOXO3-NM_001415139.1      333 VSAQN---SRRNVMLRNDPMMS-----------------------FAAQP
CC-FOXO-XM041212798.1        444 VASQN---QRQMVMCSDPAISP-----------------------FNAQS
BF-FOXO-XP_035685890.1       470 MSSGDPLISQGNLMLRQDPMMSGCPDQGSQLSSLLQGTGMSYAFRQQQQQ
DM-FOXO-NM_001275628.1       458 QCLEVLNNEAQPIDEFNLENFPVG----------------------NLEC
Ce-DAF16-AF032112.1          465 ASSSALPIDLENLTLP----------------------------------
```

```
                          601 .......610.......620.......630.......640.......650
PL-FOXO-MT799801.2        428 TSPHRLMPS-GNQGSNLAMLLNNGHN--QTTSHHHPLPYPNGGTPHHIPH
SP-FOXO1-XP_030850453     425 TSPHRLMPSSMNQGSNLAMLLNNGHNTHTTSHHHPQPYPNGGTPHHVHP
HL-FOXO-KAJ8026986.1      465 R--QRFVSPGQHQQVNQSSLALALGMS---QQGFTPASLQMSRNIQAYQQ
AR-FOXO-XP_033635399.1    467 GRSLQGFSPQTSPSNPLSGLLLPNAVT---ASQTNHMSSPTHVSLNAHYA
SK-FOXO3-NM_001164968.1   488 GNNLQPFQQQQQQQQHSRLLAQLIGGNALQQQLQQQQQQQQQQQQQQQQQ
HS-FOXO3-NM_001415139.1   357 NQGSLVNQNLLHHQHQTQGALGGSRALSNSVSNMGLSESSSLGSAKHQQQ
CC-FOXO-XM041212798.1     468 PRLMNSNPFHHPSTAQQNSAVNNGALSNPIGLMHMHSDAGNINSVAHHLQ
BF-FOXO-XP_035685890.1    520 QLQQQQQQQQPQQQPQQQNYNLGCALNQMLGGNMGIMAGQLAPTQQQQQ
DM-FOXO-NM_001275628.1    486 NVEELLQQEMSYGGLLDINIPLATVNTNLVNSSSGPLSISNISNLSNISS
Ce-DAF16-AF032112.1       481 -------------------------------DQPLMDTMDVDALIRHELS

                          651 .......660.......670.......680.......690.......700
PL-FOXO-MT799801.2        475 IHAH-HQHHHHPGIGHQDRFPSDLES-------------------VQIDPL
SP-FOXO1-XP_030850453     475 HHIHPHIHHHHPGMVHVDRFPSDLES------------------VQIDPL
HL-FOXO-KAJ8026986.1      510 QSTQQNLQSQLVPN-----GPIPNDL------------------VSIEVE
AR-FOXO-XP_033635399.1    514 QSMNQLLQSSPMTTNTSDRVPTDLQS------------------LNVDMF
SK-FOXO3-NM_001164968.1   538 QQQQQMLSNQRTNFSMLQSMNMNMNMPGLATDQLTNMHDKFPSDLEIDMF
HS-FOXO3-NM_001415139.1   407 ---SPVSQSMQTLSDSLSGSSLYSTS----ANLPVMGHEKFPSDLDIDMF
CC-FOXO-XM041212798.1     518 NQLHSLASHGMQMEASDSRLSSCPGG----INISTMSQDKFPTDLDIDMF
BF-FOXO-XP_035685890.1    570 TPLPQISTFGMMHQQSPASLHQQAMANSQANQLLSMHQEKFPSDLEIDMF
DM-FOXO-NM_001275628.1    536 NSGSSLSLNQLQAQLQQQQQQQQAQQ----------------QQQAQQQ
Ce-DAF16-AF032112.1       500 QAGGQHIHFDL---------------------------------------

                          701 .......710.......720.......730.......740.......750
PL-FOXO-MT799801.2        506 KGWSDLDVETIIRNE------------QDLTEG-PDASFDNIGTIGTTATT
SP-FOXO1-XP_030850453     507 KGWSDLDVETIIRNE------------QDLTEG-PDASFDNIGTIGTTATT
HL-FOXO-KAJ8026986.1      537 P-EFDIDMDSFIRNE------------VNLGDGFNNVNFDNINTGNPTSTA
AR-FOXO-XP_033635399.1    546 K-DMHCDIESVIHDE------------LKMDDGNLDFNFD-----GPINNQ
SK-FOXO3-NM_001164968.1   588 NGGLECDVDSIIRNEPDITETAALDFNNLEGNTSSTMGMNLTFSTSSATS
HS-FOXO3-NM_001415139.1   450 NGSLECDMESIIRSELMDAD--GLDFNFDSLISTQNVVGLNVGNFTGAKQ
CC-FOXO-XM041212798.1     564 HGSLECDVESIILNEFMDSE--ELDFNFDCAMPTQSVG-INMATLPTAPQ
BF-FOXO-XP_035685890.1    620 SGGLECDMDSIINTELMEDG--GLEFNFQQDPNNQMGSCAPTSAGMTQSM
DM-FOXO-NM_001275628.1    569 QQQHQQHQQQLLLNNNNNSS----SSLELATQTATTNLNARVQYSQPSVV
Ce-DAF16-AF032112.1           --------------------------------------------------

                          751 .......760.........
PL-FOXO-MT799801.2        544 MAAPSWVH-----------
SP-FOXO1-XP_030850453     545 MAAPSWVH-----------
HL-FOXO-KAJ8026986.1      575 NIGTNWVH-----------
AR-FOXO-XP_033635399.1    579 TVAPNWVH-----------
SK-FOXO3-NM_001164968.1   638 SSTQSWVH-----------
HS-FOXO3-NM_001415139.1   498 ASSQSWVPG----------
CC-FOXO-XM041212798.1     611 TTNQSWVPG----------
BF-FOXO-XP_035685890.1    668 TMSQTQTNAGRLGYRETVI
DM-FOXO-NM_001275628.1    615 TSPPSWVH-----------
Ce-DAF16-AF032112.1           -------------------
```
