# Supplementary material for: Gene, Protein, and in Silico Analyses of FoxO, an Evolutionary Conserved Transcription Factor in the Sea Urchin Paracentrotus lividus
Source: Genes (Basel). 2024 Aug 15;15(8):1078. doi: 10.3390/genes15081078 (PMC11353378; doi:10.3390/genes15081078)
Supplement: Supplementary file 1 [file genes-15-01078-s001.zip › Fig. S5.pdf]

Phyre2

Email	roberta.russo@irib.cnr.it
Description	F1______
Date	Sun May 19 07:33:50 BST 2024
Unique Job ID	a5a371f4c060a91e

Secondary structure and disorder prediction

	1–60
Sequence	MVDNDPDFEPQARPRSCTWPLRRPDFLDSKPQQPGNAAAAPPVDHPHGALSPAVLTEESV
Secondary structure	
SS confidence	
Disorder	??-----??
Disorder confidence	

	61–120
Sequence	DIKPILPLEGGENRELSTPSSQRRNGSRRNAWGNLSYADLITKAIQSAPDQRLTLSQIYD
Secondary structure	
SS confidence	
Disorder	???????????????????????????????????-------------------------
Disorder confidence	

	121–180
Sequence	WMVKNVPFFKDKGDSNSSAGWKNSIRHNLSLHSRFVRVQNEGTGKSSWWMINPDAKPGKS
Secondary structure	
SS confidence	
Disorder	---???
Disorder confidence	

	181–240
Sequence	SRRRASSMDTTNSKFERKRGRVKKKVLEERAKWGNTSPTPKLEGEEGASPLPFNLATTDF
Secondary structure	
SS confidence	
Disorder	??
Disorder confidence	

	241–300
Sequence	RSRASSNASSCGRLSPIMTTHPEMDMHDNEVPPMSPIPFQDIAPSQAYDSPDPYQSTDQL
Secondary structure	
SS confidence	
Disorder	??
Disorder confidence	

	301–360
Sequence	AKLAKAMTLDSSLSVEPAIRHPHNNGGYLFSPQSYSGSDMSPVHSNTQSPYYSQQGTPAV
Secondary structure	
SS confidence	
Disorder	??
Disorder confidence	

	361–420
Sequence	SPLGQCSPMQELPPNQYGMRQTFTSLMHENNDAIIPQDPMFSQTAGLRQQQSPRPMPSCR
Secondary structure	
SS confidence	
Disorder	??

Disorder confidence	

	430 · 440 · 450 · 460 · 470 · 480
Sequence	EESMIQHTSPHRLMPSGNQGSNLAMLLNNGHNQTTSHHHPLPYPNGGTPHHIPHIHAHHQ
Secondary structure	
SS confidence	
Disorder	??
Disorder confidence	

	490 · 500 · 510 · 520 · 530 · 540
Sequence	HHHHPGIGHQDRFPSDLESVQIDPLKGWSDLDVETILRNEQDLTEGPDASFDNIGTIGTT
Secondary structure	
SS confidence	
Disorder	?????????????????---------------------------------------????
Disorder confidence	

	550
Sequence	ATTMAAPSWVH
Secondary structure	
SS confidence	
Disorder	???????????
Disorder confidence	

Confidence Key

High(9) Low (0)

? Disordered (77%)

Alpha helix (8%)

Beta strand (2%)
