# Supplementary material for: Gene, Protein, and in Silico Analyses of FoxO, an Evolutionary Conserved Transcription Factor in the Sea Urchin Paracentrotus lividus
Source: Genes (Basel). 2024 Aug 15;15(8):1078. doi: 10.3390/genes15081078 (PMC11353378; doi:10.3390/genes15081078)

Figure S6 Phylogenetic tree of FoxO proteins generated by NCBI Blast program calculated with Neighbor joining, 0,85 max seq difference grishin distance of protein.

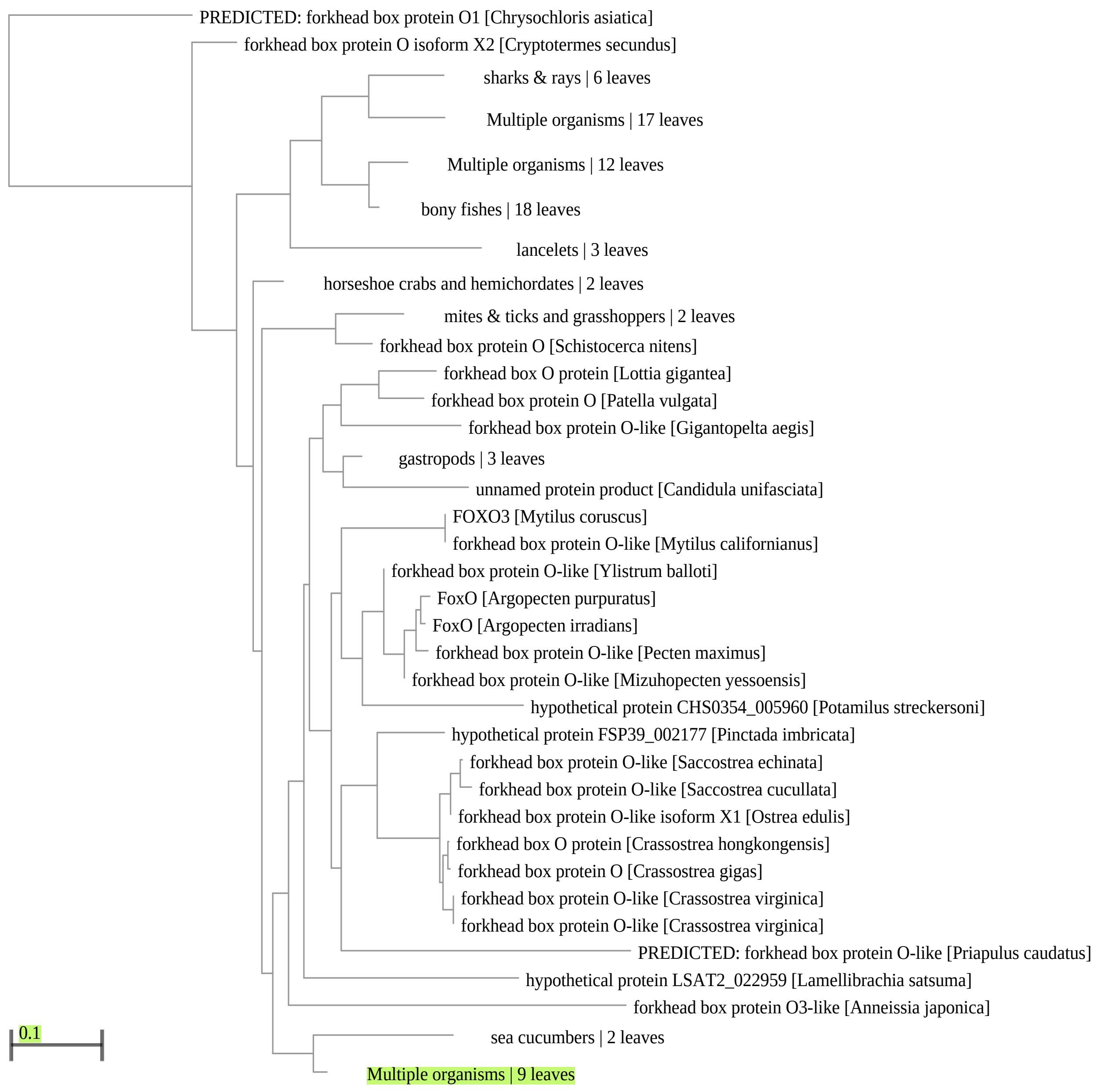

Supplement: Supplementary file 1 [file genes-15-01078-s001.zip › Fig. S6.pdf]
