# Supplementary material for: Gene, Protein, and in Silico Analyses of FoxO, an Evolutionary Conserved Transcription Factor in the Sea Urchin Paracentrotus lividus
Source: Genes (Basel). 2024 Aug 15;15(8):1078. doi: 10.3390/genes15081078 (PMC11353378; doi:10.3390/genes15081078)
Supplement: Supplementary file 1 [file genes-15-01078-s001.zip › Fig. S7.pdf]

Figure S7. Whole mount in situ hybridization (WMISH) with *Pl-foxo* probe on *P. lividus* embryos at the late gastrula stage.

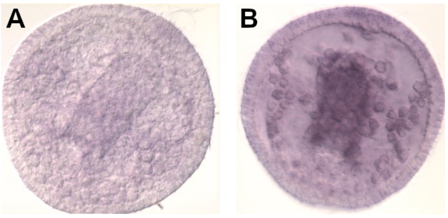

(A) Sense and (B) anti-sense *Pl-foxo* mRNA probes. WMISH was performed as previously described by Chiaramonte et al. [23] with some modifications. Hybridizations were carried out at 58 °C for 68 h and, after extensive washing, embryos were incubated with an anti-DIG alkaline phosphatase conjugated antibody (Roche Applied Science, Germany) for 3 h at room temperature. The DIG-labeled probes were detected by staining with the chromogenic 5-bromo-4-chloro-3-indolyl-phosphate and 4-toluidine/4-nitro blue tetrazolium chloride substrates (Roche). After staining, embryos were observed under a Zeiss Axioskop 2 Plus microscope and images were recorded.
