# Supplementary material for: Gene, Protein, and in Silico Analyses of FoxO, an Evolutionary Conserved Transcription Factor in the Sea Urchin Paracentrotus lividus
Source: Genes (Basel). 2024 Aug 15;15(8):1078. doi: 10.3390/genes15081078 (PMC11353378; doi:10.3390/genes15081078)
Supplement: Supplementary file 1 [file genes-15-01078-s001.zip › File S1.pdf]

*P. lividus* database: http://octopus.obs-vlfr.fr/blast/oursin/blast_oursin.php

*P. lividus* database: http://octopus.obs-vlfr.fr/blast/oursin/blast_oursin.php
